# SEPPA-mAb: spatial epitope prediction of protein antigens for mAbs

**DOI:** 10.1093/nar/gkad427

**Published:** 2023-05-22

**Authors:** Tianyi Qiu, Lu Zhang, Zikun Chen, Yuan Wang, Tiantian Mao, Caicui Wang, Yewei Cun, Genhui Zheng, Deyu Yan, Mengdi Zhou, Kailin Tang, Zhiwei Cao

**Affiliations:** School of Life Sciences, Fudan University, Shanghai 200092, China; Institute of Clinical Science, Zhongshan Hospital, Fudan University, Shanghai 200032, China; Shanghai Tenth People's Hospital, School of Life Sciences and Technology, Tongji University, Shanghai 200092, China; Shanghai Tenth People's Hospital, School of Life Sciences and Technology, Tongji University, Shanghai 200092, China; Shanghai Tenth People's Hospital, School of Life Sciences and Technology, Tongji University, Shanghai 200092, China; Shanghai Tenth People's Hospital, School of Life Sciences and Technology, Tongji University, Shanghai 200092, China; Shanghai Tenth People's Hospital, School of Life Sciences and Technology, Tongji University, Shanghai 200092, China; School of Life Sciences, Fudan University, Shanghai 200092, China; Shanghai Tenth People's Hospital, School of Life Sciences and Technology, Tongji University, Shanghai 200092, China; Shanghai Tenth People's Hospital, School of Life Sciences and Technology, Tongji University, Shanghai 200092, China; Shanghai Tenth People's Hospital, School of Life Sciences and Technology, Tongji University, Shanghai 200092, China; Shanghai Tenth People's Hospital, School of Life Sciences and Technology, Tongji University, Shanghai 200092, China; School of Life Sciences, Fudan University, Shanghai 200092, China; Shanghai Tenth People's Hospital, School of Life Sciences and Technology, Tongji University, Shanghai 200092, China

## Abstract

Identifying the exact epitope positions for a monoclonal antibody (mAb) is of critical importance yet highly challenging to the Ab design of biomedical research. Based on previous versions of SEPPA 3.0, we present SEPPA-mAb for the above purpose with high accuracy and low false positive rate (FPR), suitable for both experimental and modelled structures. In practice, SEPPA-mAb appended a fingerprints-based patch model to SEPPA 3.0, considering the structural and physic-chemical complementarity between a possible epitope patch and the complementarity-determining region of mAb and trained on 860 representative antigen-antibody complexes. On independent testing of 193 antigen-antibody pairs, SEPPA-mAb achieved an accuracy of 0.873 with an FPR of 0.097 in classifying epitope and non-epitope residues under the default threshold, while docking-based methods gave the best AUC of 0.691, and the top epitope prediction tool gave AUC of 0.730 with balanced accuracy of 0.635. A study on 36 independent HIV glycoproteins displayed a high accuracy of 0.918 and a low FPR of 0.058. Further testing illustrated outstanding robustness on new antigens and modelled antibodies. Being the first online tool predicting mAb-specific epitopes, SEPPA-mAb may help to discover new epitopes and design better mAbs for therapeutic and diagnostic purposes. SEPPA-mAb can be accessed at http://www.badd-cao.net/seppa-mab/.

## Graphical Abstract

**Graphical Abstract:**
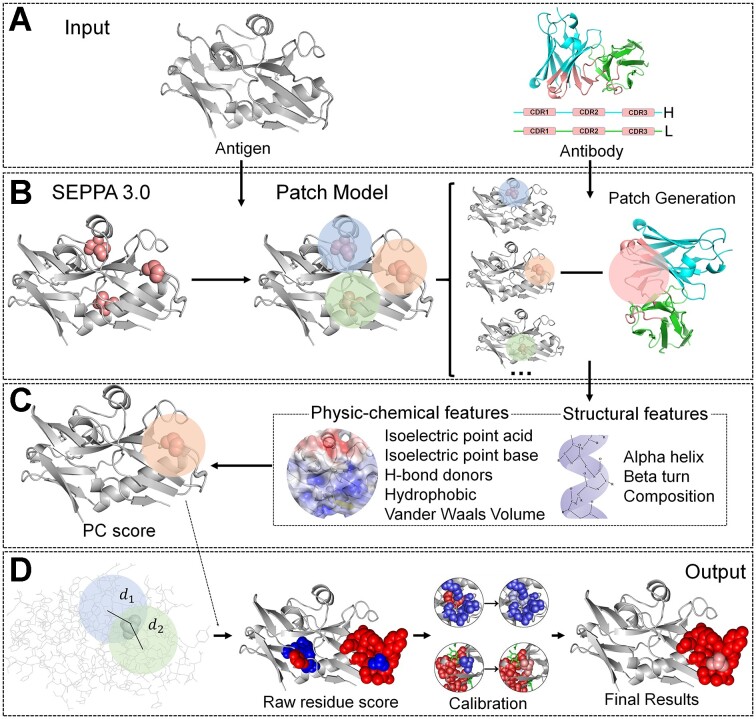

## INTRODUCTION

Monoclonal antibodies (mAbs) play important roles in adaptive immune protection, *in vitro* diagnostic, and clinical therapy owing to their capacity to specifically recognize and bind to the epitope residues in antigen protein (1). The recent development of BCR-sequencing and library screening technologies have rendered rapid mAb harvesting from the vaccinated animals (2), yet the further characterization of Ab-specific epitope positions remains highly challenging, mainly due to the intrinsic spatial nature of Ab-antigen binding (3). Though more and more online tools to predict spatial epitopes have emerged, with notable examples of Epitopia (4), CBTOPE (5), Discotope2.0 (6), BepiPred (7,8) and SEPPA 3.0 (9), they usually focused on the antigens alone, missing the information of cognate antibodies. In other words, these methods forecast all antigenic residues on the antigen surface, which may be targeted by multiple antibody clusters, instead of a specific mAb.

Meanwhile, docking-based strategies were also employed by treating the antigen-antibody interaction as a general protein-protein interaction (10,11). Typical approaches including ZDOCK (12), and ClusPro (13) calculate the complementarity between biomolecules in terms of shape, electrostatics and statistical potential for scoring. Usually, docking-based methods predict multiple possible regions without cutoff for one mAb to bind. In addition, drastically decreased accuracy was detected on modelled structures from antibody sequences (14).

Currently, a new trend has emerged in attempting to develop antibody-specific epitope predictors. For instance, Martin et al developed an antibody-specific B-cell epitope predictor based on antibody-antigen protein complexes. This method divided the surface of the antigen structures into patches and used a feed-forward neural network for model construction (15). Epipred proposed a global docking-based algorithm to identify the epitope region (10). PECAN designed an antigen-antibody interaction algorithm based on a graph convolution attention network (16). More recently, AbAdapt raised an adaptive approach to predict antibody-antigen complex structures on the sequence level (17). Xu *et al.* proposed a pipeline based on AlphaFold to integrate antibody and antigen structural modelling with rigid docking to predict antibody-specific epitopes (18). However, none of the above provided user-accessible tools or software. Here, we present SEPPA-mAb, the first online tool for predicting mAb-specific epitopes. SEPPA-mAb was composed of two models: SEPPA 3.0 (9), which calculated all potential antigenic sites based solely on antigen structure, and a fingerprint-based patch model, scoring the potential complementarity between epitope patch and complementarity-determining region (CDR) patch. The final integration generated high accuracy and a low false-positive rate, which may be useful to biomedical users.

## DATASET

Antigen-antibody structure complexes were extracted and curated with unique epitopes from Protein Database Bank (PDB) (19). Surface, epitope and paratope residues were defined as the same as the SEPPA series (9). Finally, 860 complexes deposited before July 2017 were selected as the internal training dataset (Supplementary Table S1), and 193 complexes after the date were used as the independent testing dataset (Supplementary Table S2), including 36 HIV Env glycoproteins (Supplementary Table S3).

## METHODS

The construction of SEPPA-mAb includes three steps: (i) calculating the antigenicity score of SEPPA 3.0, (ii) constructing the patch model and obtaining the patch model score at the residue level and (iii) consolidating the patch model score with the antigenicity score based on the threshold.

In step two, the design of the patch model includes four sub-steps: (i) deriving a group of surface patches for antigen, (ii) generating a series of descriptors for each surface patch of antigen and the CDR patch of the corresponding antibody, then patch complementarity (PC) score was calculated by XGBoost classifier, (iii) mapping the patch scores to each surface residue and then calculating the raw residue score by considering all the patches that contain the target residue, (iv) obtaining the final patch model score on the residue level through the calibration process. Detailed information on each step is described in Supplementary Method 1.

### Algorithm of SEPPA-mAb

For any query of an antigen-antibody structure pair, SEPPA-mAb predicts which residues on the antigen surface can bind to the CDR of the antibody through the following steps.

Step1: Generate a spatial surface patch for each surface residue on the target antigen (see ***Design of Patch Model***);

Step2: Generate the structure fingerprints for the surface patch of the antigen and CDRs patch of the antibody, and then the PC score was predicted for each surface patch and CDR patch pair (see ***Design of Patch Model***);

Step3: Map the PC scores to each surface residue to obtain the raw residue score according to Equation (1);

Step4: Calculate the patch model scores on the residue level by calibration and normalization of the raw residue scores according to Equations (2) and (3);

Step5: Consolidate the patch model scores and antigenicity scores predicted by SEPPA 3.0 to obtain the final list of mAb-specific epitope residues, when both scores are over the thresholds.

### Design of patch model

For the input antigen, SEPPA-mAb will automatically generalize the spatial patch for each surface residue on antigen protein and be paired with the CDR patch of the corresponding antibody. Then, the patch model will generate fingerprints, and calculate the patch model score on the residue level.

During fingerprint generation, the patch model introduced a cylinder model describing the structural layout and physic-chemical properties for each patch based on the defaulted pixel. Eight properties are considered to generate the 200-bit fingerprints for the antigen side and antibody side, separately. After being trained on 860 antigen-antibody pairs through XGBoost, the patch model can predict the PC score for each antigen patch according to the CDR of cognate antibody (*see Supplementary Method 1*).

To determine whether one residue is an epitope residue or not, the predicted PC scores are first mapped to the individual residue. Considering all the patches that contain the same residue, the raw residue score for any residue r can be calculated through Equation (1):


(1) $$\begin{equation*}raw\_residue\_scor{e}_r = \frac{{\sum \frac{1}{{1 + d}}*PC\_scor{e}_i}}{M}\end{equation*}$$

where $PC\_scor{e}_i$ represents the predicted PC score of surface patch *i* which contains residue r, and $d$ is the distance of residue r to the center of patch *i*, while $M$ is the total number of patches which contains residue *r*.

Then, to identify the final patch model score, calibration, and normalization were introduced for the raw residue scores. The calibration process is designed to adjust the raw residue scores of individual residues based on the overall tendency of neighboring residues. The adjusted residue score of residue *r* is defined by the averaged raw residue score of all neighboring surface residues as Equation (2) illustrated:


(2) $$\begin{equation*}adjust\_residue\_scor{e}_r = \frac{{\sum raw\_residue\_scor{e}_j}}{N}\end{equation*}$$

where $\sum raw\_residue\_scor{e}_j$ represents the sum of the raw residue score of all neighboring surface residues within 5 Å atom distance of target residue *r*, while $N$ means the total number of above residues.

Finally, the normalization process is conducted to make the results comparable between different antigens. The $adjust\_residue\_score$ was normalized to a range of 0–1 to obtain the patch model score using Equation (3):


(3) $$\begin{eqnarray*}Patch\ Model\_scor{e}_r = \frac{{adjust\_residue\_scor{e}_r - min\left( {adjust\_residue\_score} \right)}}{{max\left( {adjust\_residue\_score} \right) - min\left( {adjust\_residue\_score} \right)}}\nonumber\\ \end{eqnarray*}$$

where $min( {adjust\_residue\_score} )$ is the minimum $adjust\_residu{\rm{e}}\_score$ of residues in a given antigen, and $max( {adjust\_residue\_score} )$ means the maximum $adjust\_residue\_score$ of residues in a given antigen. $Patch{\rm{\ }}Model\_scor{e}_r$ means the patch model score for a specific residue *r*.

## RESULTS

### Patch model construction and performance test

On top of the antigenic sites predicted by SEPPA 3.0, we develop a patch model to evaluate the physic-chemical complementarity of possible contacting regions between the antigen surface and CDR surface by reporting a patch model score between 0 and 1. The evaluation parameters of the area under the ROC curve (AUC) value and balanced accuracy (BA) are adopted as illustrated in SEPPA 3.0 (9). For internal validation, eight machine learning approaches are screened, including XGBoost (XGB), Support Vector Machine (SVM), Random Forrest (RF), Decision Tree (DT), Multi-Layer Perceptron (MLP), Gradient Descent (GD), Gaussian Naïve Bayes (GNB) and Linear Regression (LR) were evaluated through 5-folds cross-validation on 860 protein structures in the training dataset. The validation indicates that XGBoost (XGB) give the best prediction results with an Area Under ROC Curve (AUC) value of 0.776, which outperforms all others. Thus, XGBoost (XGB) is chosen to construct the patch model.

**Figure 1.**
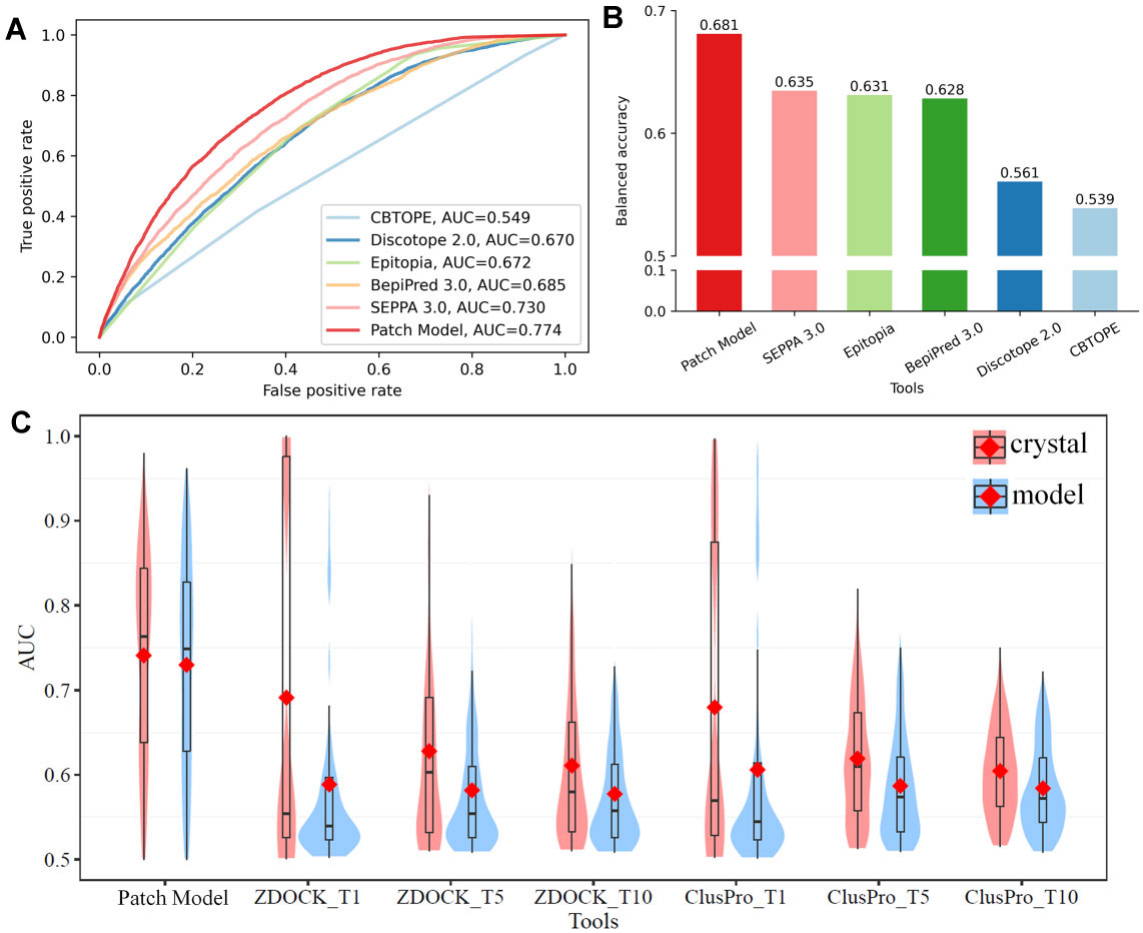
Performance comparison between patch model and available state-of-the-art methods. (**A**) ROC curves for traditional epitope prediction methods on independent test dataset containing 193 antigens. (**B**) Balanced accuracy for epitope prediction methods on independent test dataset containing 193 antigens. (**C**) AUC distribution in violin plot on test dataset containing 193 antigens for patch model, ZDOCK and ClusPro respectively. The red dot represented the averaged AUC value for different methods. T1, T5 and T10 represent the top 1, top 5 and top 10 output lists from docking approaches respectively over the test dataset containing 193 antigens. The results obtained by experimental structures and modelled structures were colored in red and blue respectively.

The performance of the patch model is tested on 193 antigens and compared with available tools online including both well-known epitope prediction methods and docking-based methods via AUC and Balanced Accuracy (BA) value. As Figure 1 shows, 7 tools are selected for comparison including 5 traditional epitope prediction tools of Epitopia (4), CBTOPE (5), DiscoTope2.0 (6), BepiPred3.0 (8) and SEPPA 3.0 (9), and two docking-based methods of ZDOCK (12) and ClusPro (13). The test dataset containing 193 antigens was not overlapping with the training dataset of any above state-of-the-art methods (Figure 1A). Results illustrate that the patch model gives the best results with an AUC of 0.774 and BA of 0.681 based on the default threshold. SEPPA 3.0 (9) achieve the second best among all current state-of-the-art methods with an AUC value of 0.730 and BA of 0.635, followed by Bepipred 3.0 with an AUC value of 0.685 and BA of 0.628(Figure 1B).

Further, two representative docking-based tools, ZDOCK (12) and ClusPro (13) are included for comparison with the patch model on both experimental and simulated structures of antibodies (*see Supplementary Method 2 for details*). Since docking approaches output multiple complex structures, the AUC values of top *N* ranking results are calculated based on combining residues from N regions. When the results are checked between experimental and modelled antibody structures by ABodyBuilder (20) (see *Supplementary Method 3* and Supplementary Table S7), docking methods indeed display an obvious performance drop on modelled structures, agreeing with previous reports (14). As illustrated in Figure 1c, the overall AUC value of the top 1 prediction decreased from 0.691 to 0.589 for ZDOCK (12) and 0.680 to 0.601 for ClusPro (13), respectively. A similar drop in the top 5 and top 10 results. Among 193 predictions, the patch model gave better AUC prediction on 122 (63%) data points than the top 1 prediction of ZDOCK and 120 (62%) data points than the top 1 results of ClusPro (Supplementary Table S4).

In terms of the patch model, it outperforms ZDOCK (12) and ClusPro (13) on different levels of top *N* comparison (Figure 1C). In addition, the patch model seems to show the ability of stable performance with an overall AUC of 0.741 on 193 crystalized, and an AUC of 0.730 on 193 simulated Ab structures. Further among the 193 modelled structures, the patch model owns better AUC values on 150 (78%) antigens than the top 1 prediction of ZDOCK, and 145 (75%) antigens than the top 1 results of ClusPro (Supplementary Table S5), demonstrating its unique ability to tolerate structure variation.

### Performance and case study of SEPPA-mAb tool

**Figure 2.**
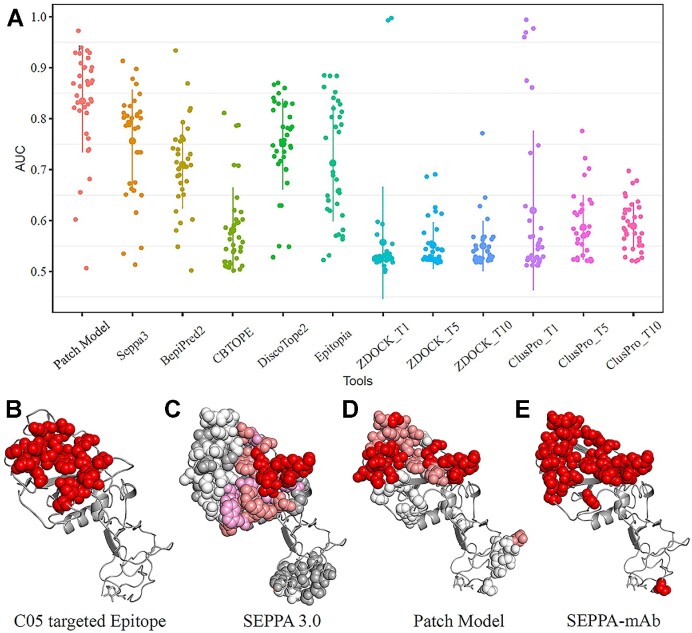
Performance and case study of SEPPA-mAb. (**A**) AUC values for patch and current state-of-the-art methods on the independent testing dataset of 36 gp120 antigens. T1, T5, and T10 represent the average AUC value of the top 1, top 5 and top 10 solutions for docking approaches respectively. (**B**) Reference epitope targeted by antibody C05 (PDB ID: 6D0U, Chain: G). (**C**) Predicted epitope residues for HA by SEPPA 3.0 with the gradient illustration under default cutoff. Red, salmon, pink, white, and grey color illustrate those candidates from high score to low score. (**D**) Predicted epitope residues for HA by Patch Model with gradient illustration under default cutoff. Red, salmon and white colors illustrate those candidates from high scores to low scores. (**E**) Combined epitope residues by incorporating SEPPA 3.0 with Patch Model.

The patch model is designed to calculate the complementarity score of interacting surfaces between the antigen and its cognate antibody, while SEPPA 3.0 is designed to score the antigenic sites on the antigen surface. Both scores are normalized from 0 to 1. For each residue, SEPPA-mAb simply considers the two scores and gives a judgment of YES (1 for epitope) if both scores are above their default cutoffs, otherwise NO (−1 for non-epitope) or NOT AVAILABLE (0 for internal residue). In this way, the prediction accuracy on the test dataset containing 193 antigens is 0.790 for the patch model and 0.776 for SEPPA 3.0, with an FPR of 0.196 for the patch model and 0.206 for SEPPA 3.0, respectively. After integration, SEPPA-mAb significantly pushes the accuracy to 0.873 and reduced FPR to 0.097. We also examined the well-known HIV glycoproteins in the test dataset containing 193 antigens, as they represent the largest family of pathogenic antigens. Patch model alone achieves the averaged AUC value of 0.835 on the 36 antigens of gp120 (Supplementary Table S3), higher than the best traditional epitope prediction tool of SEPPA 3.0 (AUC = 0.756) and the best docking approach of ClusPro (AUC < 0.65 for Top 1, Top 5 and Top 10 solutions) (Figure 2A). Further integration achieves an advanced accuracy of 0.918 and an FPR of 0.058 for SEPPA-mAb.

To better illustrate the benefits to integrate the patch model with SEPPA 3.0, the reference epitope in hemagglutinin (HA) antigen targeted by antibody C05 (PDB ID: 6D0U, Chain: G) was shown in Figure 2B. Results showed that SEPPA 3.0 predicts many spreading antigenic sites (Figure 2C), while the patch model suggests two major regions relatively complemented to antibody C05 (Figure 2D). The final integration of SEPPA-mAb efficiently removes those false positive residues, presenting the best candidate positions as C05-targeted epitopes (Figure 2E).

The pandemic of COVID-19 in 2020 provided an opportunity to test model performance on completely new antigens never seen by SEPPA-mAb. Here, 31 pairs of spike antigens and their cognate Abs were tested with detailed PDB ID listed in Supplementary Table S6. The average accuracy of 0.753 with an average FPR of 0.224 is achieved by SEPPA-mAb. As the accuracy is calculated at the individual residue level which is highly stringent, we examined whether SEPPA-mAb can predict the correct epitope area for each antibody (30% residue overlapping between predicted and crystalized epitope positions). The result shows our model can successfully suggest 23 out of 31 epitope areas, indicating the outstanding ability on completely new antigens.

## USAGE

### Input

SEPPA-mAb (http://www.badd-cao.net/seppa-mab) accepts input files of antigen-antibody pair in the below format: (i) existing PDB IDs with chain name, and (ii) local files in PDB format. Similar to SEPPA 3.0 (9), users are recommended to select subcellular localization of protein antigen and species of immune host if available. Also, batch query submission is encouraged. During the batch query, users can submit multiple entries including specified PDB IDs, subcellular localization, species of immune host, and chain name. After successful submission, each residue of antigen protein will be processed by patch model with the information of the corresponding antibody to calculate a score of possible Ab interaction, and by SEPPA 3.0 to obtain the antigenicity score respectively.

### Output

**Figure 3.**
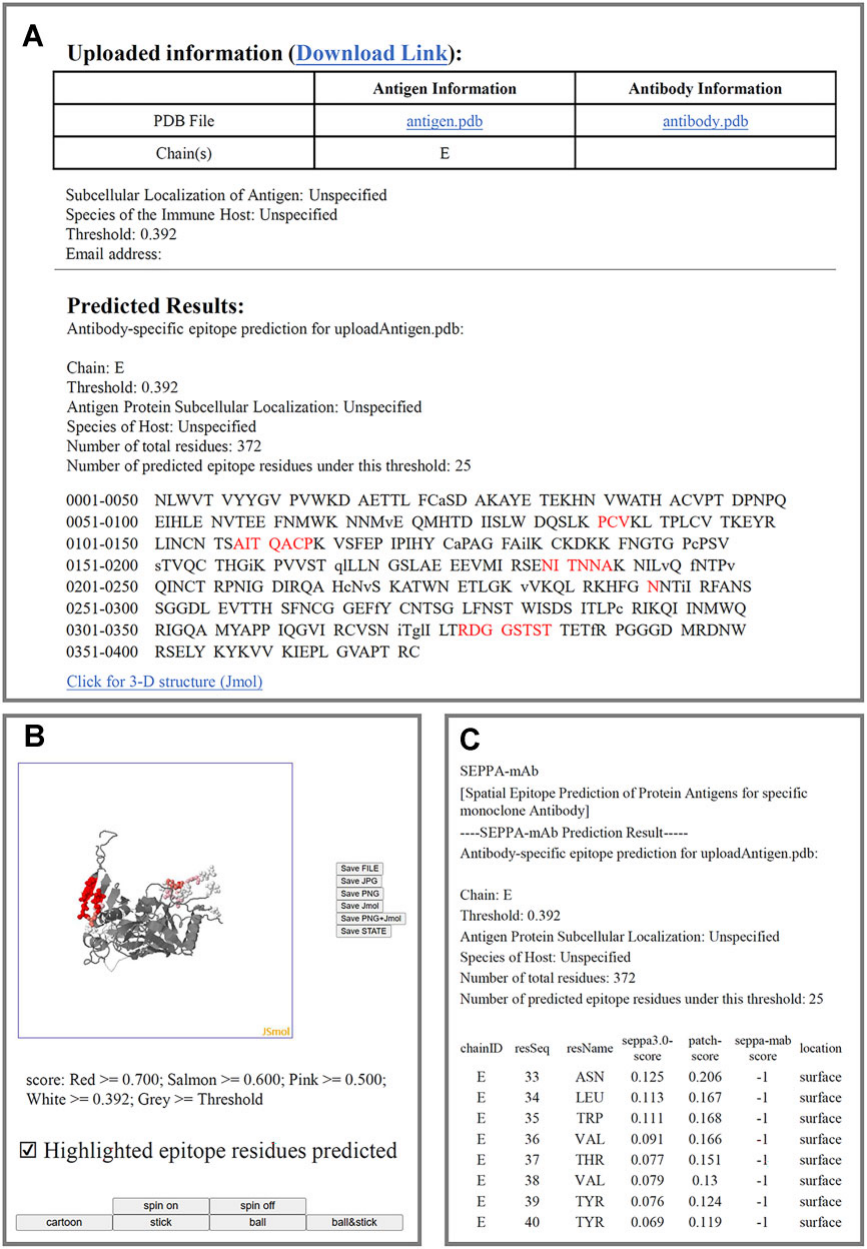
Illustration of SEPPA-mAb output results. (**A**) Result summary for epitope prediction of query antigen. (**B**) 3D visualization of query antigen. (**C**) Results predicted for each residue in queried antigen including scores from SEPPA 3.0, patch model score, and SEPPA-mAb.

The output results of SEPPA-mAb will either be presented in .html format by browsing the progress of calculation via job-id or sent back to users via email. The .html format will provide a result summary from three aspects: (i) submission information, including model parameters, sequence of antigen, and sequence of antibody, as well as the predicted epitope information, including the residue positions of the input antigen. Predicted epitope residues are presented in red capital letters and the non-surface amino acids are presented in lowercase letters (Figure 3A); (ii) epitope 3-D visualization, facilitating users to observe the spatial distribution of epitopes. The 3D model of the antigen is created via Jmol. The amino acid is labelled in different colors based on the SEPPA-mAb scores (Figure 3B) and (iii) downloadable results in .txt format, which includes the query information, SEPPA 3.0 score, patch model score, and SEPPA-mAb score for each residue (Figure 3C). More information can be found on the HELP page of SEPPA-mAb.

## DISCUSSION

Predicting the Ab-specific epitopes for protein antigens is highly desired but no tool is available online. In this study, we developed SEPPA-mAb for this purpose, based on the previous antigenicity prediction server of SEPPA 3.0, appended by a new patch model calculating the physic-chemical complementarity between antigen-antibody interaction surface. Compared with current state-of-the-art methods of traditional epitope prediction tools and docking-based algorithms, the patch model alone shows outstanding performance on both Ab-specific interface prediction and robustness on varied structure variation. More importantly, the integrated tool of SEPPA-mAb can efficiently increase the accuracy and reduce the FPR, with the best ability to tolerate structure variation of computer modelling.

Prediction B-cell epitopes for a cognate binding or neutralizing antibody have received increasing attention in recent years. As more and more antibodies are derived from one antigen, it is becoming apparent that essentially different surface regions of an antigen may be recognized and bound by antibodies (21,22). Missing the information of a cognate antibody, what those traditional algorithms (4,7,9) calculate are actually kind of pan-antigenic sites. Then several pioneers reformulated the question from predicting pan-antigenic sites to mAb-specific epitope suggestion (15,22). For instance, Rapberger et al proposed that the antigen epitope should geometrically and electrostatically match the antibody structure (23). More recently, the Ab-specific epitope predictor by Martin *et al.* divided the antigen surface residues into multiple patches to generate 471 features including 237 for antigen patch and 234 for antibody paratope (15). Though no accessible tools are provided to biomedical users, the above brought enlightening guidance for the development of SEPPA-mAb.

The performance of SEPPA-mAb mainly benefits from both the successful prediction of potential antibody-complimentary regions from the patch model, and the pan-antigenic sites predicted by SEPPA 3.0. Importantly, our patch model is designed with several novelty aspects. The first is the patch-based structural and physicochemical fingerprints derived from the cylinder model. Via cylinder (*Supplementary Method 1*), the layers of local micro-environmental variations can be fully considered for a surface residue under the neighborhood influence of both surface and internal residues. Secondly, different from the previous patch conception where all residues in the patch are equally treated, we give patch residues weighted scaling according to their distance to the patch center. In this way, the residual layout and subsequent physic-chemical properties can be well captured from both sides of the interaction interface describing the complex nature of antigen-antibody interactions. Thirdly, all calculation is made on the residue level, instead of the detailed atom coordinates in docking methods. This coarse-grained description enables rapid surface scanning patch-pairing, also tolerating structural variation caused by computer modelling. Finally, a calibration process is elaborated to further reduce FPR by considering the neighboring influence. In summary, SEPPA-mAb consolidated the results from pan-antigenic sites predicted by SEPPA 3.0, and mAb CDR-complementary surface predicted by patch model, enhancing the prediction performance from baseline (accuracy of 0.776–0.790, FPR of 0.196–0.206) to a level with high accuracy of 0.873 and low FPR of 0.097.

Be noted that, the current model aims to recommend the best epitope positions in antigen surface being recognized by its cognate antibodies. Any input antibody is regarded as interacting with input antigen by expectation. Mechanistically, SEPPA-mAb employed more information from antigens rather than antibodies, leading to its insensitivity to antibodies. In fact, it is more sensitive to antigen mutation and structural variation. As SEPPA-mAb conducts the calculation based on the structure files, incomplete structures may reduce their performance. Further, SEPPA-mAb considered the influence of glycosylation through SEPPA 3.0. Other forms of post-translational modifications such as phosphorylation, ubiquitination, methylation, and so on, have not been considered in the current version. Also, single-chain antibodies are not applicable for now. In the future, with the rapid accumulation of structures generated through experiments and AI technologies, as well as the development of deep learning algorithms, improved versions can be expected for antibody-specific epitope prediction, which may better assist antibody design in therapeutic and diagnostic purposes.

## DATA AVAILABILITY

The data underlying this article are available in the article and in its online supplementary material. Accession number PDB ID: 6D0U.

## Supplementary Material

gkad427_Supplemental_File
