# Supplementary material for: SEPPA-mAb: spatial epitope prediction of protein antigens for mAbs
Source: Nucleic Acids Res. 2023 May 22;51(W1):W528–34. doi: 10.1093/nar/gkad427 (PMC10320061; doi:10.1093/nar/gkad427)
Supplement: gkad427_Supplemental_File [file gkad427_supplemental_file.docx]

**Supplementary Table**

**Supplementary Table 1. Training dataset**

| **PDB ID and Chain name^a^** | | | | |
| --- | --- | --- | --- | --- |
| 1A14_N_HL | 2ZCH_P_HL | 4D9R_A_HL | 4RRP_N_HB | 5J3H_E_CD |
| 1A2Y_C_BA | 2ZCK_P_HL | 4DAG_A_HL | 4RWY_A_HL | 5JQ6_A_HL |
| 1ADQ_A_HL | 2ZCL_P_HL | 4DGI_A_HL | 4RX4_G_HL | 5JW3_B_HL |
| 1AFV_A_HL | 2ZJS_Y_HL | 4DKE_A_HL | 4S1Q_G_HL | 5JXE_B_DC |
| 1AHW_C_BA | 3A67_Y_HL | 4DKF_A_HL | 4S1R_G_HL | 5JZ7_B_HL |
| 1AR1_B_CD | 3A6B_Y_HL | 4DN4_M_HL | 4S1S_G_HL | 5K59_B_EF |
| 1BVK_C_BA | 3A6C_Y_HL | 4DQO_C_HL | 4TNV_P_Wf | 5K9K_F_HL |
| 1CZ8_W_HL | 3AB0_A_BC | 4DTG_K_HL | 4TSA_A_HL | 5K9O_I_HL |
| 1DEE_G_DC | 3B2U_A_HL | 4DVR_G_HL | 4TSB_A_HL | 5KAQ_B_HL |
| 1DQJ_C_BA | 3B9K_B_HL | 4DW2_U_HL | 4TSC_A_HL | 5KEL_A_CD |
| 1E6J_P_HL | 3BDY_V_HL | 4EDW_V_HL | 4U0R_A_BC | 5KEL_I_QU |
| 1EGJ_A_HL | 3BE1_A_HL | 4EDX_V_HL | 4U1G_A_BC | 5KEM_A_DE |
| 1EO8_A_HL | 3BGF_S_HL | 4ETQ_C_HL | 4U6H_E_AB | 5KEM_A_BC |
| 1FBI_X_HL | 3BN9_B_DC | 4F15_J_KL | 4U6V_B_KM | 5KEN_B_CD |
| 1FDL_Y_HL | 3BT2_U_HL | 4F2M_E_AB | 4UAO_A_CB | 5KEN_K_QP |
| 1FE8_A_HL | 3C09_D_HL | 4F3F_C_BA | 4UT6_B_IM | 5KJR_G_HL |
| 1FJ1_F_BA | 3C09_A_CB | 4FFV_A_HL | 4UT9_D_JN | 5KQV_F_PQ |
| 1FNS_A_HL | 3CSY_J_AB | 4FFW_B_DC | 4UTA_B_HL | 5KVD_E_HL |
| 1FSK_G_IH | 3CSY_L_EF | 4FFY_A_HL | 4UTB_A_HL | 5KVE_E_HL |
| 1G7H_C_BA | 3CXH_E_JK | 4FP8_A_HL | 4UU9_D_HL | 5KVF_E_HL |
| 1G7I_C_BA | 3D85_C_BA | 4FQJ_A_HL | 4V1D_C_AB | 5KVG_E_HL |
| 1G7J_C_BA | 3D9A_C_HL | 4FQK_C_EF | 4WFF_A_ED | 5KW9_A_HL |
| 1G7L_C_BA | 3DVG_X_BA | 4FQR_A_ab | 4WV1_C_BA | 5KZC_A_HL |
| 1G7M_C_BA | 3DVG_Y_BA | 4FQV_D_JN | 4XAK_A_HL | 5L0Q_A_CB |
| 1GC1_G_HL | 3DVN_U_HL | 4FQY_B_HL | 4XMN_E_HL | 5L6Y_C_HL |
| 1H0D_C_BA | 3DVN_X_BA | 4G3Y_C_HL | 4XMP_G_HL | 5LCV_A_HL |
| 1HEZ_E_DC | 3EFF_M_BA | 4G6J_A_HL | 4XNM_C_HL | 5LQB_A_HL |
| 1HEZ_E_BA | 3EHB_B_CD | 4G6M_A_HL | 4XNQ_D_BA | 5LSP_A_HL |
| 1I9R_A_HL | 3EO1_I_HG | 4G7V_S_HL | 4XNX_A_HL | 5LWY_A_HL |
| 1IC4_Y_HL | 3EOA_I_HL | 4G7Y_S_HL | 4XNY_G_HL | 5LXG_A_HL |
| 1IC7_Y_HL | 3F7V_C_AB | 4G80_I_GH | 4XNZ_D_EF | 5MES_A_HL |
| 1IQD_C_BA | 3FB5_C_AB | 4GMS_A_HL | 4XNZ_G_HL | 5MEV_A_HL |
| 1J1O_Y_HL | 3FB8_C_AB | 4GXU_A_MN | 4XP1_A_HL | 5MHR_E_PO |
| 1J1P_Y_HL | 3G04_C_BA | 4H88_A_HL | 4XP4_A_HL | 5MI0_A_BC |
| 1J1X_Y_HL | 3G6D_A_HL | 4H8W_G_HL | 4XP5_A_HL | 5MO9_X_HL |
| 1JHL_A_HL | 3G6J_D_HG | 4HC1_A_HL | 4XP6_A_HL | 5MVZ_U_HL |
| 1JPS_T_HL | 3GBM_B_HL | 4HCR_A_HL | 4XPA_A_HL | 5N09_A_HL |
| 1JRH_I_HL | 3GBN_B_HL | 4HF5_A_HL | 4XPH_A_HL | 5N0A_A_HL |
| 1KB5_B_HL | 3GI8_C_HL | 4HFU_A_HL | 4XRC_C_HL | 5N7W_X_HL |
| 1KEN_C_TU | 3GI9_C_HL | 4HG4_D_PQ | 4XTR_A_EF | 5NGV_A_HL |
| 1KIP_C_BA | 3GRW_A_HL | 4HJ0_B_CD | 4XVS_G_HL | 5NH3_A_HL |
| 1KIR_C_BA | 3H42_B_HL | 4HJG_H_BA | 4XVT_G_HL | 5NHR_C_HL |
| 1KYO_E_JK | 3HB3_B_CD | 4HJG_E_BA | 4XWG_A_HL | 5NJ6_A_HL |
| 1LK3_B_IM | 3HFM_Y_HL | 4HKX_E_AB | 4XWO_N_QR | 5NJG_A_EF |
| 1MHP_A_HL | 3HI1_G_HL | 4HKZ_E_BA | 4XWO_M_OP | 5NMV_K_HL |
| 1MLC_E_BA | 3HI6_A_HL | 4HLZ_D_IJ | 4XX1_A_HL | 5NUZ_D_HL |
| 1N6Q_B_HL | 3HMX_A_HL | 4HT1_T_HL | 4XZU_G_EF | 5O14_A_HL |
| 1N8Z_C_BA | 3I50_E_HL | 4HWB_A_HL | 4Y5V_C_AB | 5O1R_A_HL |
| 1NBY_C_BA | 3IDX_G_HL | 4I18_R_AB | 4Y5X_I_GH | 5O4G_C_BA |
| 1NBZ_C_BA | 3IDY_A_BC | 4I18_C_HL | 4Y5Y_C_AB | 5O6V_B_HL |
| 1NCA_N_HL | 3IU3_I_HL | 4I2X_E_BA | 4YBL_A_BC | 5OB5_A_HL |
| 1NCB_N_HL | 3IYW_A_HL | 4I2X_F_DC | 4YBQ_B_DC | 5OCC_A_HL |
| 1NCC_N_HL | 3J5M_E_HG | 4I3R_G_HL | 4YC2_G_HL | 5SX4_N_HL |
| 1NDG_C_BA | 3J70_P_MN | 4I3S_G_HL | 4YC2_A_BC | 5SX5_N_HL |
| 1NDM_C_BA | 3JWO_A_HL | 4I77_Z_HL | 4YDI_G_HL | 5SY8_O_HL |
| 1NFD_B_FE | 3K2U_A_HL | 4IDJ_A_HL | 4YDJ_G_HL | 5T33_G_HL |
| 1NFD_D_HG | 3KJ4_A_HL | 4IJ3_A_CB | 4YDK_G_HL | 5T3S_G_HL |
| 1NMA_N_HL | 3KJ6_A_HL | 4IOF_B_EF | 4YDL_A_BC | 5T3X_G_DE |
| 1NMB_N_HL | 3KLH_B_DC | 4IRZ_A_HL | 4YE4_G_HL | 5T3Z_G_HL |
| 1NSN_S_HL | 3KR3_D_HL | 4J4P_A_HL | 4YFL_G_HL | 5T3Z_G_DE |
| 1OAK_A_HL | 3KS0_A_KJ | 4J6R_G_HL | 4YFL_E_FI | 5T5B_O_AB |
| 1OAZ_A_HL | 3L5W_I_HL | 4JAN_G_HL | 4YK4_A_CB | 5T5F_A_HL |
| 1OB1_C_BA | 3L5X_A_HL | 4JB9_G_HL | 4YPG_D_HL | 5T6L_I_HL |
| 1ORQ_C_BA | 3L95_Y_HL | 4JDT_G_HL | 4YWG_G_HL | 5T80_G_HL |
| 1ORS_C_BA | 3LDB_A_CB | 4JHW_F_HL | 4YX2_A_HL | 5T85_G_HL |
| 1OSP_O_HL | 3LH2_V_KO | 4JKP_G_HL | 4YXH_A_HL | 5TE4_G_HL |
| 1P2C_C_BA | 3LHP_S_HL | 4JLR_S_HL | 4YXK_A_HL | 5TE6_G_HL |
| 1PKQ_J_GF | 3LIZ_A_HL | 4JM2_E_DC | 4YXL_A_HL | 5TE7_G_HL |
| 1QFU_A_HL | 3LQA_G_HL | 4JPK_A_HL | 4YZF_A_EF | 5TFW_O_HL |
| 1QFW_B_IM | 3LQA_C_HL | 4JPV_G_HL | 4Z5R_X_ZY | 5TH9_B_IM |
| 1QLE_B_HL | 3LZF_A_HL | 4JPW_G_HL | 4ZFG_A_HL | 5THR_B_TU |
| 1RJL_C_BA | 3MA9_A_HL | 4JR9_A_HL | 4ZPT_R_AB | 5THR_E_NK |
| 1RZK_G_HL | 3MAC_A_HL | 4JRE_D_HL | 4ZPT_S_HL | 5TIH_A_HL |
| 1S78_B_FE | 3MJ9_A_HL | 4JZJ_C_HL | 4ZPV_R_HL | 5TL5_A_HL |
| 1SY6_A_HL | 3MXW_A_HL | 4K24_U_HL | 4ZS6_A_HL | 5TLJ_X_DC |
| 1TZH_W_BA | 3N85_A_HL | 4K2U_A_HL | 4ZS7_A_HL | 5TLJ_X_BA |
| 1UA6_Y_HL | 3NCY_B_PS | 4K3J_B_HL | 4ZSO_F_DC | 5TLK_X_BA |
| 1UAC_Y_HL | 3NFP_I_HL | 4K8R_B_DC | 4ZXB_E_CD | 5TLK_X_DC |
| 1UJ3_C_BA | 3NGB_A_BC | 4K8R_B_HL | 4ZXB_E_AB | 5TPN_A_HL |
| 1V7M_V_HL | 3NH7_A_HL | 4K9E_C_HL | 4ZYP_A_KM | 5TPW_A_HL |
| 1V7N_X_IM | 3NID_A_HL | 4KHT_A_HL | 4ZYP_B_NO | 5TQ0_A_HL |
| 1VFB_C_BA | 3NPS_A_BC | 4KHX_A_HL | 5A7X_E_HG | 5TQ2_A_HL |
| 1WEJ_F_HL | 3O2D_A_HL | 4KI5_M_EF | 5ACO_A_HL | 5TQQ_A_HL |
| 1XCQ_M_DC | 3OGC_C_AB | 4KRO_A_DC | 5ANM_G_BA | 5TR1_B_IM |
| 1XCT_L_BA | 3OPZ_A_HL | 4KRP_A_DC | 5B3J_D_HL | 5TRU_c_hl |
| 1XCT_M_DC | 3P0Y_A_HL | 4KV5_B_JI | 5B71_F_DC | 5TUD_A_CB |
| 1XF5_L_BA | 3P11_A_HL | 4KVN_A_HL | 5B8C_I_HG | 5TZ2_C_HL |
| 1XGQ_C_BA | 3PGF_A_HL | 4KXZ_B_JI | 5B8C_C_BA | 5TZU_C_HL |
| 1XGR_C_BA | 3PJS_M_BA | 4L5F_E_HL | 5BO1_B_HL | 5U7O_G_HL |
| 1XGT_C_BA | 3PNW_C_BA | 4LEO_C_AB | 5BV7_A_HL | 5U8Q_A_HL |
| 1XIW_E_HG | 3Q1S_I_HL | 4LF3_F_ED | 5BV7_A_CB | 5U8R_A_HL |
| 1YJD_C_HL | 3Q3G_G_DC | 4LIQ_E_HL | 5BVP_I_HL | 5UCB_B_HL |
| 1YMH_F_DC | 3QA3_E_BA | 4LMQ_D_HL | 5C0R_A_HL | 5UDC_F_HL |
| 1YNT_E_BA | 3QUM_P_BA | 4LQF_A_HL | 5C0S_A_HL | 5UEA_D_HL |
| 1YNT_G_DC | 3QUM_Q_KM | 4LSP_G_HL | 5C6T_A_HL | 5UEK_A_HL |
| 1YQV_Y_HL | 3QWO_P_HL | 4LSQ_G_HL | 5C7X_A_HL | 5UEM_G_HL |
| 1YY9_A_DC | 3R08_E_HL | 4LSR_G_HL | 5C8J_I_AB | 5UG0_A_DC |
| 1YYL_P_RQ | 3R1G_B_HL | 4LSS_G_HL | 5CBA_E_AB | 5UGY_A_HL |
| 1YYM_P_RQ | 3RAJ_A_HL | 4LST_G_HL | 5CBE_F_CD | 5UKR_G_HL |
| 1Z3G_A_HL | 3RHW_B_FN | 4LSU_G_HL | 5CD5_A_CD | 5UM8_G_HL |
| 1ZA3_R_HL | 3RKD_A_HL | 4LSV_G_HL | 5CEZ_G_HL | 5UMN_A_EF |
| 1ZTX_E_HL | 3RU8_X_HL | 4LU5_B_HL | 5CJO_A_HL | 5UOE_C_PQ |
| 2A0L_B_FE | 3RVW_A_DC | 4LVH_A_BC | 5CJQ_B_HL | 5USH_X_HL |
| 2ADF_A_HL | 3S35_X_HL | 4LVN_A_CB | 5CUS_C_JN | 5USL_X_HL |
| 2AEP_A_HL | 3S36_X_HL | 4LVO_A_CB | 5CZV_A_HL | 5UTZ_E_FG |
| 2AEQ_A_HL | 3S37_X_HL | 4M1C_A_CD | 5CZX_A_HL | 5V2A_A_HL |
| 2ARJ_Q_HL | 3SDY_B_HL | 4M1G_B_HL | 5D1Q_E_CD | 5V7J_G_HL |
| 2ATK_C_AB | 3SE8_G_HL | 4M1G_A_HL | 5D1Q_E_BA | 5V8L_A_HL |
| 2B2X_A_HL | 3SE9_G_HL | 4M5Z_A_HL | 5D1X_E_BA | 5V8M_G_SU |
| 2B4C_G_HL | 3SKJ_E_HL | 4M62_T_IM | 5D1X_E_CD | 5VAG_A_CB |
| 2BDN_A_HL | 3SOB_B_HL | 4M7L_T_HL | 5D1Z_I_DC | 5VEB_X_AB |
| 2CMR_A_HL | 3SQO_A_HL | 4M8Q_S_AB | 5D1Z_J_GH | 5VGJ_G_HL |
| 2DD8_S_HL | 3T2N_A_HL | 4M8Q_C_HL | 5D72_B_MN | 5VIC_E_HL |
| 2DQC_Y_HL | 3T3M_C_EF | 4MA7_A_HL | 5D8J_A_HL | 5VIG_Z_HL |
| 2DQE_Y_HL | 3TT1_A_IM | 4MHH_E_HL | 5D93_A_CB | 5VJO_E_AB |
| 2DQF_C_BA | 3TT3_A_HL | 4MHJ_A_HL | 5D96_A_CB | 5VJQ_J_CD |
| 2DQG_Y_HL | 3U2S_G_HL | 4MWF_D_AB | 5D9Q_A_FE | 5VKD_A_HL |
| 2DQJ_Y_HL | 3U30_A_CB | 4MXV_A_HL | 5DFV_A_CD | 5VKE_C_AB |
| 2EIZ_C_BA | 3U4E_G_HL | 4MXW_A_HL | 5DHV_N_AB | 5VL3_Q_HL |
| 2FD6_U_HL | 3U7Y_G_HL | 4NHH_B_MK | 5DHZ_M_HL | 5VL7_B_HL |
| 2FJH_V_HL | 3U9P_D_HL | 4NNP_A_HL | 5DMI_A_HL | 5VLP_A_HL |
| 2H9G_S_HL | 3U9U_F_AB | 4NP4_A_IM | 5DO2_A_HL | 5VN3_J_MO |
| 2I5Y_P_RQ | 3UAJ_A_HL | 4NP4_A_HL | 5DUM_A_HL | 5VOB_D_HL |
| 2I9L_K_FE | 3UBX_A_HL | 4NZT_M_HL | 5DUP_A_HL | 5VOC_D_HL |
| 2IFF_Y_HL | 3UC0_A_HL | 4O02_A_HL | 5DUR_C_HL | 5VPL_A_DC |
| 2J6E_A_HL | 3ULU_A_DC | 4O58_A_HL | 5DWU_A_HL | 5VTA_D_HL |
| 2J88_A_HL | 3ULU_A_FE | 4O5I_K_WX | 5E1A_C_AB | 5VYF_F_HL |
| 2JEL_P_HL | 3ULU_A_HL | 4O9H_A_HL | 5E8E_H_BA | 5W06_T_HL |
| 2JIX_E_DG | 3ULV_A_DC | 4OD2_S_BA | 5E94_G_BA | 5W08_C_KL |
| 2JIX_C_FA | 3ULV_A_FE | 4ODX_X_HB | 5EII_G_HL | 5W0D_A_BC |
| 2LTQ_A_CB | 3ULV_A_HL | 4OGA_E_CD | 5EN2_C_AB | 5W0K_C_EF |
| 2NR6_B_FE | 3V4P_B_HL | 4OGX_A_HL | 5EU7_B_FD | 5W0K_A_HL |
| 2NY3_A_DC | 3V4V_B_HL | 4OGY_B_MN | 5EZO_A_HL | 5W1K_P_ON |
| 2NY4_A_DC | 3V6O_B_DF | 4OGY_A_HL | 5F3B_D_EF | 5W1K_J_LK |
| 2NY5_G_HL | 3V6Z_F_AB | 4OII_A_HL | 5F3H_I_CD | 5W1M_T_DC |
| 2NY7_G_HL | 3V7A_A_EH | 4OKV_F_AB | 5F6J_E_HF | 5W23_C_HL |
| 2NZ9_A_DC | 3VG9_A_CB | 4OLU_G_HL | 5F96_G_HL | 5W2B_A_HL |
| 2OZ4_A_HL | 3VGA_A_CB | 4OLV_G_HL | 5F9O_G_HL | 5W3E_B_EG |
| 2Q8A_A_HL | 3VI3_D_HL | 4OLW_G_HL | 5F9W_G_HL | 5W3L_B_EG |
| 2Q8B_A_HL | 3VI4_D_HL | 4OLX_G_HL | 5FB8_C_BA | 5W3M_B_EG |
| 2QAD_A_DC | 3VRL_C_HL | 4OLY_G_HL | 5FCU_G_HL | 5W3O_B_DE |
| 2QQK_A_HL | 3W11_E_CD | 4OLZ_G_HL | 5FEC_I_HL | 5W42_A_HL |
| 2QQL_A_HL | 3W2D_A_HL | 4OM0_G_HL | 5FHC_K_HL | 5W5X_A_HL |
| 2QQN_A_HL | 3W9E_C_AB | 4OM1_G_HL | 5FYL_B_DE | 5W5Z_A_HL |
| 2QR0_P_RQ | 3WD5_A_HL | 4OQT_A_HL | 5FYL_G_HL | 5W6D_G_HL |
| 2R0K_A_HL | 3WFB_C_HL | 4OT1_A_HL | 5G64_A_HL | 5W6G_A_HL |
| 2R0L_A_HL | 3WIH_A_HL | 4P59_A_HL | 5GGR_Z_HL | 5W9H_D_EF |
| 2R29_A_HL | 3WKM_A_HL | 4PLJ_A_HL | 5GGS_Y_CD | 5W9I_I_KL |
| 2R4R_A_HL | 3WLW_B_HL | 4PLK_A_HL | 5GGT_A_HL | 5W9J_A_BC |
| 2R4S_A_HL | 3WXV_A_HL | 4PP1_B_DC | 5GGV_Y_HL | 5W9K_G_HI |
| 2R56_A_HL | 3WXW_A_HL | 4PP2_E_DC | 5GJS_B_HL | 5W9L_G_HI |
| 2R69_A_HL | 3X3F_A_HL | 4PS4_A_HL | 5GJT_B_HL | 5W9M_A_BC |
| 2UZI_R_HL | 3ZDY_C_EF | 4PY8_A_IJ | 5GMQ_A_BC | 5W9N_A_BC |
| 2VDP_A_HL | 3ZDZ_C_EF | 4PY8_B_IJ | 5GRJ_A_HL | 5W9O_A_BC |
| 2VIR_C_BA | 3ZE0_A_HL | 4Q6I_C_HL | 5GS0_A_DC | 5W9P_B_DE |
| 2VIS_C_BA | 3ZE1_A_HL | 4QCI_C_HL | 5GZO_A_HL | 5WB9_G_HL |
| 2VIT_C_BA | 3ZE2_C_HL | 4QEX_A_HL | 5H35_D_HI | 5WT9_G_HL |
| 2VWE_A_EC | 3ZKM_A_HL | 4QHU_C_HL | 5H37_A_GH | 5WUX_E_HL |
| 2VXQ_A_HL | 3ZKN_A_HL | 4QTI_U_HL | 5HBT_B_DC | 5X0T_E_AB |
| 2VXT_I_HL | 3ZTJ_B_GH | 4QWW_B_DC | 5HBV_B_DC | 5X8L_B_GL |
| 2W9E_A_HL | 4AEI_A_HL | 4R0L_D_HL | 5HDQ_A_HL | 5X8M_A_BC |
| 2WUB_C_RQ | 4AG4_A_HL | 4R8W_B_HL | 5HHV_A_HL | 5XBM_C_BA |
| 2WUC_A_HL | 4AL8_C_HL | 4RAU_C_BA | 5HJ3_L_MN | 5XEZ_B_HL |
| 2X7L_M_GI | 4ALA_C_HL | 4RDQ_E_GF | 5HYS_G_AB | 5XF1_B_HL |
| 2XQB_A_HL | 4BZ1_A_HL | 4RFN_G_HL | 5I5K_B_HL | 5XHV_E_HL |
| 2XQY_A_GL | 4BZ2_A_HL | 4RFO_G_HL | 5I8H_A_IJ | 5XJ3_L_JK |
| 2XRA_A_HL | 4CAD_F_ED | 4RGM_A_CB | 5I9Q_G_HL | 5XJ4_A_HL |
| 2XTJ_A_DB | 4CKD_C_HL | 4RGM_S_HL | 5IES_C_HL | 5XJM_A_HL |
| 2XWT_C_AB | 4CMH_A_BC | 4RGN_A_DE | 5IF0_I_AB | 5XKU_A_CB |
| 2YC1_C_AB | 4CNI_C_HL | 4RGN_A_BC | 5IGX_G_HL | 5XMH_A_HL |
| 2YPV_A_HL | 4D3C_A_HL | 4RGO_S_HL | 5IKC_M_BA | 5XS7_A_HL |
| 2YSS_C_BA | 4D9Q_A_HL | 4RQS_G_DC | 5J13_A_CB | 5XWD_A_HD |

^a^ Each code represents the information of PDB id (4 letter code), antigen chain (one letter code) and antibody chain (two letter code). For example, for 1A14_N_HL, 1A14 represents the PDB id, N represents the antigen chain and HL represents the antibody chain.

**Supplementary Table 2. Testing dataset**

| **PDB ID and Chain name^a^** | | | | |
| --- | --- | --- | --- | --- |
| 5BJZ_B_DH | 6B0S_C_HL | 6CK9_G_HL | 6FAX_R_HL | 6MEK_C_FE |
| 5BK1_A_HL | 6B3S_A_HL | 6CM3_A_RS | 6FEQ_A_FE | 6MEK_A_BD |
| 5BK2_A_HL | 6B70_B_EF | 6CM3_E_ON | 6FGB_A_HL | 6MFT_G_HL |
| 5OTJ_C_HL | 6B7Z_B_EF | 6CMG_A_CB | 6FLA_I_HL | 6MHR_C_AB |
| 5WDU_G_DE | 6B9J_X_HL | 6CMI_B_DC | 6FLB_G_HL | 6MI2_C_AB |
| 5WHK_A_HL | 6BAE_E_BA | 6CMO_A_HL | 6FLC_G_HL | 6ML8_A_HL |
| 5WI9_A_HL | 6BAH_C_BA | 6CNV_B_HL | 6FN1_A_CB | 6MLK_A_HL |
| 5WOB_G_UV | 6BCK_G_HL | 6CRQ_A_DE | 6FN4_A_CB | 6MLM_A_EI |
| 5XXY_A_HL | 6BDZ_A_HL | 6CSE_M_HL | 6FXN_A_DE | 6MTO_T_HL |
| 5Y11_C_AB | 6BF7_B_EF | 6CSF_M_HL | 6FY1_G_HL | 6MTQ_T_HL |
| 5Y2L_B_IJ | 6BF9_A_CD | 6CUE_C_QR | 6GV1_A_HL | 6MU6_G_HL |
| 5Y9J_A_HL | 6BFQ_K_HL | 6CUF_d_qr | 6H3T_B_IM | 6MU7_G_HL |
| 5YOY_K_PM | 6BFS_C_HL | 6CUF_d_mn | 6H3U_B_HL | 6MU7_B_DE |
| 5YWY_A_HL | 6BFT_G_AB | 6CW2_D_AB | 6H5N_A_CB | 6MU8_G_HL |
| 5YY5_A_HL | 6BFT_C_HL | 6CW3_H_AB | 6HCO_B_DC | 6MUF_G_HL |
| 5ZXV_A_HL | 6BGT_C_BA | 6CWT_F_CD | 6HF1_A_CB | 6MUG_G_HL |
| 6A0Z_A_HL | 6BIT_G_JL | 6CXY_C_HL | 6I04_A_HL | 6MUI_B_CD |
| 6A3W_C_AB | 6BKB_E_HL | 6CYF_A_DC | 6I8S_D_HL | 6MW9_B_CD |
| 6A4K_B_IM | 6BKC_E_HL | 6D0U_G_HI | 6I9I_D_HL | 6MWC_B_CD |
| 6A67_A_HL | 6BKD_E_HL | 6D2P_C_HL | 6IEA_A_HL | 6MWV_J_KL |
| 6A78_A_HL | 6BP2_A_HL | 6D6T_B_JI | 6IEB_A_HL | 6MWX_N_OP |
| 6AL5_A_HL | 6BPA_A_BC | 6D6U_B_JI | 6IEC_A_HL | 6MWX_J_KL |
| 6AOD_C_BA | 6BPC_A_BC | 6DDM_C_BA | 6IEK_A_BC | 6MYY_E_GF |
| 6APB_C_HL | 6BPE_D_EF | 6DDR_C_BA | 6II4_A_HL | 6NB3_C_HL |
| 6APD_C_JL | 6C5V_A_HL | 6DDV_C_BA | 6II8_C_HL | 6NM6_G_HL |
| 6APD_C_FG | 6C6Y_R_HL | 6DE7_G_HL | 6II9_C_MN | 6NM6_G_UV |
| 6AQ7_A_HL | 6C6Z_B_HL | 6DFI_E_HL | 6IUT_A_HL | 6NN3_A_HL |
| 6ARU_A_CB | 6C9U_A_HL | 6DFJ_E_HL | 6IUV_B_CD | 6NNF_G_UV |
| 6ATT_A_HL | 6CBV_B_HL | 6DID_G_KE | 6IVZ_A_HL | 6NNF_G_HL |
| 6AYZ_A_BD | 6CDE_C_QR | 6E3H_B_HL | 6IW2_J_KL | 6NNJ_G_UV |
| 6AZ2_B_AF | 6CDE_C_MN | 6E62_A_BC | 6J5D_A_HL | 6O39_C_BA |
| 6AZZ_A_CB | 6CDI_C_QR | 6E63_P_HL | 6J5G_A_HL | 6O3A_E_BA |
| 6B08_A_CB | 6CE0_B_DE | 6EAY_A_HL | 6MAR_B_MN | 6O3B_H_GE |
| 6B0A_A_HL | 6CF2_F_AB | 6EDU_D_LM | 6MDT_G_HL | 6QEE_A_CB |
| 6B0E_E_BA | 6CH7_B_DE | 6EDU_F_PQ | 6MDT_B_DE | 6QEX_A_CB |
| 6B0G_E_DC | 6CHB_H_OP | 6ELU_D_EF | 6MEH_C_HL | 6R2S_C_BA |
| 6B0H_I_DC | 6CHB_G_JK | 6ETI_A_FE | 6MEI_C_HL | 6R8X_A_CB |
| 6B0N_G_HL | 6CHB_G_DE | 6EWB_C_GI | 6MEJ_C_AB |  |
| 6B0N_G_DE | 6CHB_F_MN | 6EYO_A_HL | 6MEJ_C_HL |  |

^a^ Each code represents the information of PDB id (4 letter code), antigen chain (one letter code) and antibody chain (two letter code). For example, for 5BJZ_B_DH, 5BJZ represents the PDB id, B represents the antigen chain and DH represents the antibody chain.

**Supplementary Table 3. Performance of Patch model compared with popular peers on HIV glycoprotein**

| PDB ID^a^ | Patch Model | SEPPA3.0 | BepiPred2 | CBTOPE | Discotope2 | Epitopia | Cluspro^b^ | ZDOCK^c^ |
| --- | --- | --- | --- | --- | --- | --- | --- | --- |
| 5WDU_G_DE | 0.829 | 0.835 | 0.74 | 0.502 | 0.803 | 0.57 | 0.6 | 0.536 |
| 6B0N_G_DE | 0.821 | 0.806 | 0.714 | 0.511 | 0.861 | 0.623 | 0.522 | 0.511 |
| 6B0N_G_HL | 0.909 | 0.848 | 0.731 | 0.587 | 0.83 | 0.835 | 0.994 | 0.993 |
| 6BCK_G_HL | 0.929 | 0.898 | 0.677 | 0.595 | 0.871 | 0.564 | 0.978 | 0.533 |
| 6CDE_C_MN | 0.834 | 0.793 | 0.648 | 0.621 | 0.743 | 0.789 | 0.512 | 0.531 |
| 6CDE_C_QR | 0.919 | 0.826 | 0.793 | 0.51 | 0.78 | 0.695 | 0.969 | 0.998 |
| 6CDI_C_QR | 0.9 | 0.793 | 0.781 | 0.512 | 0.768 | 0.654 | 0.529 | 0.52 |
| 6CE0_B_DE | 0.761 | 0.616 | 0.759 | 0.544 | 0.549 | 0.633 | 0.548 | 0.54 |
| 6CH7_B_DE | 0.875 | 0.801 | 0.87 | 0.509 | 0.711 | 0.852 | 0.555 | 0.535 |
| 6CHB_G_JK | 0.74 | 0.734 | 0.595 | 0.518 | 0.629 | 0.688 | 0.528 | 0.53 |
| 6CHB_H_OP | 0.737 | 0.734 | 0.731 | 0.563 | 0.735 | 0.523 | 0.523 | 0.53 |
| 6CHB_F_MN | 0.812 | 0.782 | 0.702 | 0.519 | 0.785 | 0.61 | 0.513 | 0.531 |
| 6CHB_G_DE | 0.816 | 0.65 | 0.705 | 0.575 | 0.746 | 0.569 | 0.519 | 0.522 |
| 6CK9_G_HL | 0.884 | 0.878 | 0.687 | 0.542 | 0.784 | 0.82 | 0.52 | 0.524 |
| 6CM3_E_ON | 0.973 | 0.805 | 0.742 | 0.709 | 0.528 | 0.573 | 0.532 | 0.533 |
| 6CM3_A_RS | 0.869 | 0.651 | 0.759 | 0.508 | 0.85 | 0.803 | 0.512 | 0.597 |
| 6CRQ_A_DE | 0.934 | 0.793 | 0.763 | 0.54 | 0.834 | 0.658 | 0.513 | 0.526 |
| 6CUE_C_QR | 0.929 | 0.868 | 0.816 | 0.539 | 0.817 | 0.681 | 0.527 | 0.523 |
| 6DE7_G_HL | 0.874 | 0.788 | 0.662 | 0.617 | 0.748 | 0.762 | 0.521 | 0.525 |
| 6DID_G_KE | 0.507 | 0.514 | 0.503 | 0.515 | 0.757 | 0.649 | 0.861 | 0.518 |
| 6EDU_D_LM | 0.655 | 0.734 | 0.721 | 0.646 | 0.55 | 0.531 | 0.551 | 0.53 |
| 6EDU_F_PQ | 0.681 | 0.535 | 0.651 | 0.558 | 0.701 | 0.712 | 0.622 | 0.503 |
| 6MAR_B_MN | 0.602 | 0.662 | 0.934 | 0.811 | 0.717 | 0.885 | 0.57 | 0.594 |
| 6MDT_G_HL | 0.823 | 0.81 | 0.689 | 0.708 | 0.867 | 0.863 | 0.518 | 0.525 |
| 6MFT_G_HL | 0.94 | 0.811 | 0.765 | 0.59 | 0.826 | 0.62 | 0.568 | 0.555 |
| 6MU6_G_HL | 0.845 | 0.786 | 0.709 | 0.567 | 0.726 | 0.774 | 0.961 | 0.522 |
| 6MU7_G_HL | 0.894 | 0.825 | 0.711 | 0.553 | 0.736 | 0.784 | 0.875 | 0.523 |
| 6MU7_B_DE | 0.771 | 0.546 | 0.82 | 0.548 | 0.7 | 0.813 | 0.585 | 0.573 |
| 6MU8_G_HL | 0.901 | 0.814 | 0.706 | 0.597 | 0.725 | 0.803 | 0.748 | 0.5 |
| 6MUF_G_HL | 0.901 | 0.781 | 0.618 | 0.788 | 0.841 | 0.884 | 0.733 | 0.526 |
| 6MUG_G_HL | 0.867 | 0.675 | 0.58 | 0.786 | 0.83 | 0.886 | 0.524 | 0.522 |
| 6NM6_G_HL | 0.838 | 0.673 | 0.602 | 0.602 | 0.674 | 0.828 | 0.526 | 0.524 |
| 6NM6_G_UV | 0.87 | 0.803 | 0.703 | 0.526 | 0.782 | 0.582 | 0.544 | 0.525 |
| 6NNF_G_HL | 0.831 | 0.659 | 0.549 | 0.602 | 0.63 | 0.84 | 0.527 | 0.525 |
| 6NNF_G_UV | 0.93 | 0.913 | 0.756 | 0.519 | 0.83 | 0.66 | 0.629 | 0.527 |
| 6NNJ_G_UV | 0.864 | 0.801 | 0.674 | 0.505 | 0.758 | 0.64 | 0.562 | 0.518 |
| Average^d^ | 0.835 | 0.757 | 0.71 | 0.582 | 0.751 | 0.713 | 0.62 | 0.557 |

^a^ Each code represents the information of PDB id (4 letter code), antigen chain (one letter code) and antibody chain (two letter code). For example, for 1A14_N_HL, 1A14 represents the PDB id, N represents the antigen chain and HL represents the antibody chain.

^b^ The performance is calculated by the top 1 ranking of Cluspro.

^c^ The performance is calculated by the top 1 ranking of ZDOCK.

^d^ Average representing the averaged AUC value of 36 structures predicted by each algorithm.

**Supplementary Table 4. Performance of Patch model compared with popular peers on 193 testing datasets based on original antibody structure**

| Original^a^ | Patch Model | ZDOCK_T1 | ZDOCK_T5 | ZDOCK_T10 | ClusPro_T1 | ClusPro_T5 | ClusPro_T10 |
| --- | --- | --- | --- | --- | --- | --- | --- |
| 5bjz_B_D_H | 0.66 | 0.90 | 0.85 | 0.83 | 0.63 | 0.61 | 0.61 |
| 5bk1_A_H_L | 0.53 | 0.99 | 0.89 | 0.87 | 0.86 | 0.79 | 0.72 |
| 5bk2_A_H_L | 0.60 | 1.00 | 0.76 | 0.68 | 0.53 | 0.61 | 0.57 |
| 5otj_C_H_L | 0.74 | 1.00 | 0.67 | 0.66 | 0.86 | 0.61 | 0.60 |
| 5wdu_G_D_E | 0.83 | 0.54 | 0.52 | 0.52 | 0.60 | 0.65 | 0.64 |
| 5whk_A_H_L | 0.80 | 0.99 | 0.95 | 0.78 | 0.88 | 0.71 | 0.67 |
| 5wi9_A_H_L | 0.87 | 0.99 | 0.62 | 0.57 | 0.51 | 0.61 | 0.58 |
| 5wob_G_U_V | 0.87 | 0.51 | 0.51 | 0.51 | 0.51 | 0.51 | 0.55 |
| 5xxy_A_H_L | 0.70 | 0.82 | 0.63 | 0.59 | 0.95 | 0.69 | 0.65 |
| 5y11_C_A_B | 0.57 | 1.00 | 0.62 | 0.57 | 0.54 | 0.74 | 0.65 |
| 5y2l_B_I_J | 0.77 | 0.53 | 0.68 | 0.68 | 0.55 | 0.56 | 0.55 |
| 5y9j_A_H_L | 0.67 | 0.98 | 0.65 | 0.62 | 0.54 | 0.56 | 0.56 |
| 5yoy_K_P_M | 0.78 | 0.99 | 0.74 | 0.66 | 0.86 | 0.82 | 0.75 |
| 5ywy_A_H_L | 0.83 | 0.55 | 0.55 | 0.54 | 0.53 | 0.69 | 0.64 |
| 5yy5_A_H_L | 0.80 | 0.54 | 0.54 | 0.54 | 0.54 | 0.71 | 0.67 |
| 5zxv_A_H_L | 0.85 | 0.54 | 0.55 | 0.58 | 0.85 | 0.64 | 0.64 |
| 6a0z_A_H_L | 0.58 | 0.52 | 0.61 | 0.56 | 0.99 | 0.62 | 0.58 |
| 6a3w_C_A_B | 0.73 | 0.58 | 0.58 | 0.56 | 0.57 | 0.69 | 0.64 |
| 6a4k_B_I_M | 0.76 | 0.51 | 0.54 | 0.54 | 0.55 | 0.53 | 0.64 |
| 6a67_A_H_L | 0.73 | 1.00 | 0.74 | 0.65 | 1.00 | 0.70 | 0.63 |
| 6a78_A_H_L | 0.68 | 0.97 | 0.90 | 0.85 | 0.97 | 0.71 | 0.65 |
| 6al5_A_H_L | 0.51 | 0.59 | 0.57 | 0.56 | 0.58 | 0.61 | 0.66 |
| 6aod_C_B_A | 0.52 | 1.00 | 0.67 | 0.65 | 0.77 | 0.71 | 0.67 |
| 6apb_C_H_L | 0.69 | 0.52 | 0.63 | 0.57 | 0.52 | 0.52 | 0.52 |
| 6apd_C_F_G | 0.86 | 0.52 | 0.52 | 0.52 | 0.51 | 0.51 | 0.56 |
| 6apd_C_J_L | 0.85 | 0.52 | 0.53 | 0.53 | 0.52 | 0.61 | 0.57 |
| 6aq7_A_H_L | 0.54 | 0.52 | 0.63 | 0.63 | 0.86 | 0.79 | 0.73 |
| 6aru_A_C_B | 0.97 | 1.00 | 0.70 | 0.61 | 0.97 | 0.61 | 0.57 |
| 6att_A_H_L | 0.64 | 0.52 | 0.61 | 0.57 | 0.52 | 0.52 | 0.52 |
| 6ayz_A_B_D | 0.61 | 0.98 | 0.87 | 0.79 | 0.86 | 0.75 | 0.66 |
| 6az2_B_A_F | 0.71 | 0.54 | 0.56 | 0.60 | 0.56 | 0.57 | 0.61 |
| 6azz_A_C_B | 0.77 | 0.99 | 0.85 | 0.75 | 0.98 | 0.64 | 0.61 |
| 6b08_A_C_B | 0.74 | 0.97 | 0.70 | 0.76 | 0.96 | 0.63 | 0.61 |
| 6b0a_A_H_L | 0.59 | 0.99 | 0.76 | 0.69 | 0.88 | 0.68 | 0.63 |
| 6b0e_E_B_A | 0.53 | 0.73 | 0.71 | 0.71 | 0.93 | 0.65 | 0.64 |
| 6b0g_E_D_C | 0.53 | 0.98 | 0.72 | 0.72 | 0.98 | 0.68 | 0.65 |
| 6b0h_I_D_C | 0.75 | 0.81 | 0.75 | 0.75 | 0.53 | 0.67 | 0.63 |
| 6b0n_G_D_E | 0.82 | 0.51 | 0.61 | 0.56 | 0.52 | 0.57 | 0.59 |
| 6b0n_G_H_L | 0.91 | 0.99 | 0.61 | 0.56 | 0.99 | 0.63 | 0.57 |
| 6b0s_C_H_L | 0.77 | 0.52 | 0.68 | 0.65 | 0.53 | 0.65 | 0.68 |
| 6b3s_A_H_L | 0.76 | 0.98 | 0.93 | 0.87 | 0.97 | 0.72 | 0.68 |
| 6b70_B_E_F | 0.82 | 0.52 | 0.51 | 0.55 | 0.95 | 0.60 | 0.56 |
| 6b7z_B_E_F | 0.85 | 0.51 | 0.51 | 0.51 | 0.93 | 0.68 | 0.60 |
| 6b9j_X_H_L | 0.54 | 0.99 | 0.65 | 0.63 | 0.86 | 0.67 | 0.66 |
| 6bae_E_B_A | 0.93 | 0.88 | 0.71 | 0.69 | 0.58 | 0.56 | 0.60 |
| 6bah_C_B_A | 0.89 | 0.60 | 0.64 | 0.65 | 0.59 | 0.74 | 0.67 |
| 6bck_G_H_L | 0.93 | 0.53 | 0.69 | 0.64 | 0.98 | 0.64 | 0.63 |
| 6bdz_A_H_L | 0.87 | 0.63 | 0.55 | 0.54 | 0.53 | 0.54 | 0.56 |
| 6bf7_B_E_F | 0.80 | 0.51 | 0.51 | 0.51 | 0.51 | 0.60 | 0.58 |
| 6bf9_A_C_D | 0.87 | 0.51 | 0.51 | 0.51 | 1.00 | 0.62 | 0.57 |
| 6bfq_K_H_L | 0.63 | 0.99 | 0.75 | 0.68 | 0.59 | 0.70 | 0.63 |
| 6bfs_C_H_L | 0.81 | 0.99 | 0.93 | 0.86 | 0.89 | 0.68 | 0.68 |
| 6bft_C_H_L | 0.88 | 1.00 | 0.67 | 0.74 | 0.91 | 0.73 | 0.67 |
| 6bft_G_A_B | 0.91 | 0.99 | 0.88 | 0.78 | 0.99 | 0.70 | 0.68 |
| 6bgt_C_B_A | 0.85 | 0.61 | 0.71 | 0.67 | 0.51 | 0.59 | 0.55 |
| 6bit_G_J_L | 0.87 | 0.91 | 0.90 | 0.75 | 0.92 | 0.74 | 0.67 |
| 6bkb_E_H_L | 0.76 | 0.99 | 0.71 | 0.66 | 0.90 | 0.67 | 0.69 |
| 6bkc_E_H_L | 0.66 | 1.00 | 0.71 | 0.67 | 0.85 | 0.73 | 0.69 |
| 6bkd_E_H_L | 0.83 | 0.57 | 0.66 | 0.66 | 0.51 | 0.60 | 0.58 |
| 6bp2_A_H_L | 0.60 | 0.99 | 0.72 | 0.73 | 0.58 | 0.63 | 0.60 |
| 6bpa_A_B_C | 0.77 | 0.99 | 0.62 | 0.57 | 0.53 | 0.53 | 0.53 |
| 6bpc_A_B_C | 0.79 | 0.53 | 0.53 | 0.53 | 0.53 | 0.60 | 0.61 |
| 6bpe_D_E_F | 0.63 | 0.53 | 0.63 | 0.58 | 0.53 | 0.62 | 0.61 |
| 6c5v_A_H_L | 0.78 | 0.53 | 0.62 | 0.67 | 0.52 | 0.54 | 0.57 |
| 6c6y_R_H_L | 0.85 | 0.99 | 0.82 | 0.75 | 0.59 | 0.78 | 0.71 |
| 6c6z_B_H_L | 0.88 | 0.58 | 0.72 | 0.70 | 0.87 | 0.70 | 0.66 |
| 6c9u_A_H_L | 0.81 | 0.77 | 0.65 | 0.63 | 0.67 | 0.57 | 0.55 |
| 6cbv_B_H_L | 0.62 | 0.56 | 0.67 | 0.62 | 0.57 | 0.63 | 0.61 |
| 6cde_C_M_N | 0.83 | 0.53 | 0.53 | 0.53 | 0.51 | 0.52 | 0.53 |
| 6cde_C_Q_R | 0.92 | 1.00 | 0.62 | 0.57 | 0.97 | 0.69 | 0.65 |
| 6cdi_C_Q_R | 0.90 | 0.52 | 0.52 | 0.57 | 0.53 | 0.61 | 0.63 |
| 6ce0_B_D_E | 0.76 | 0.54 | 0.54 | 0.55 | 0.55 | 0.55 | 0.55 |
| 6cf2_F_A_B | 0.60 | 0.96 | 0.86 | 0.77 | 0.65 | 0.63 | 0.63 |
| 6ch7_B_D_E | 0.88 | 0.53 | 0.58 | 0.57 | 0.56 | 0.57 | 0.57 |
| 6chb_F_M_N | 0.81 | 0.53 | 0.53 | 0.53 | 0.51 | 0.63 | 0.61 |
| 6chb_G_D_E | 0.82 | 0.52 | 0.52 | 0.52 | 0.52 | 0.57 | 0.55 |
| 6chb_G_J_K | 0.74 | 0.53 | 0.53 | 0.52 | 0.53 | 0.52 | 0.52 |
| 6chb_H_O_P | 0.74 | 0.53 | 0.52 | 0.53 | 0.52 | 0.53 | 0.53 |
| 6ck9_G_H_L | 0.88 | 0.52 | 0.52 | 0.53 | 0.52 | 0.56 | 0.57 |
| 6cm3_A_R_S | 0.87 | 0.60 | 0.63 | 0.63 | 0.51 | 0.62 | 0.59 |
| 6cm3_E_O_N | 0.97 | 0.53 | 0.69 | 0.77 | 0.53 | 0.59 | 0.60 |
| 6cmg_A_C_B | 0.62 | 0.95 | 0.68 | 0.66 | 0.52 | 0.61 | 0.60 |
| 6cmi_B_D_C | 0.51 | 0.90 | 0.60 | 0.57 | 0.55 | 0.62 | 0.60 |
| 6cmo_A_H_L | 0.55 | 0.53 | 0.66 | 0.63 | 0.53 | 0.53 | 0.53 |
| 6cnv_B_H_L | 0.69 | 0.58 | 0.62 | 0.60 | 0.56 | 0.54 | 0.54 |
| 6crq_A_D_E | 0.93 | 0.53 | 0.52 | 0.52 | 0.51 | 0.52 | 0.62 |
| 6cse_M_H_L | 0.81 | 0.52 | 0.52 | 0.52 | 0.52 | 0.52 | 0.52 |
| 6csf_M_H_L | 0.80 | 0.52 | 0.52 | 0.53 | 0.52 | 0.52 | 0.52 |
| 6cue_C_Q_R | 0.93 | 0.52 | 0.52 | 0.52 | 0.53 | 0.62 | 0.59 |
| 6cuf_d_m_n | 0.89 | 0.52 | 0.52 | 0.52 | 0.51 | 0.60 | 0.56 |
| 6cuf_d_q_r | 0.90 | 0.52 | 0.52 | 0.52 | 0.56 | 0.60 | 0.64 |
| 6cw2_D_A_B | 0.59 | 0.99 | 0.73 | 0.65 | 0.87 | 0.71 | 0.65 |
| 6cw3_H_A_B | 0.54 | 0.96 | 0.66 | 0.63 | 0.68 | 0.58 | 0.62 |
| 6cwt_F_C_D | 0.65 | 0.56 | 0.55 | 0.55 | 0.81 | 0.60 | 0.57 |
| 6cxy_C_H_L | 0.67 | 0.54 | 0.64 | 0.63 | 0.55 | 0.65 | 0.61 |
| 6cyf_A_D_C | 0.66 | 0.56 | 0.54 | 0.59 | 0.88 | 0.81 | 0.73 |
| 6d0u_G_H_I | 0.94 | 0.54 | 0.65 | 0.59 | 0.53 | 0.54 | 0.54 |
| 6d2p_C_H_L | 0.83 | 0.77 | 0.57 | 0.60 | 0.76 | 0.75 | 0.69 |
| 6d6t_B_J_I | 0.65 | 0.54 | 0.53 | 0.53 | 0.52 | 0.64 | 0.59 |
| 6d6u_B_J_I | 0.64 | 0.52 | 0.53 | 0.53 | 0.95 | 0.61 | 0.57 |
| 6ddm_C_B_A | 0.73 | 0.60 | 0.60 | 0.57 | 0.59 | 0.67 | 0.63 |
| 6ddr_C_B_A | 0.60 | 0.76 | 0.80 | 0.70 | 0.64 | 0.60 | 0.61 |
| 6ddv_C_B_A | 0.85 | 0.99 | 0.80 | 0.74 | 0.94 | 0.63 | 0.60 |
| 6de7_G_H_L | 0.87 | 0.52 | 0.53 | 0.53 | 0.52 | 0.52 | 0.52 |
| 6dfi_E_H_L | 0.65 | 0.58 | 0.57 | 0.63 | 0.95 | 0.67 | 0.63 |
| 6dfj_E_H_L | 0.78 | 0.59 | 0.58 | 0.63 | 0.61 | 0.68 | 0.63 |
| 6did_G_K_E | 0.51 | 0.52 | 0.52 | 0.52 | 0.86 | 0.59 | 0.56 |
| 6e3h_B_H_L | 0.85 | 0.54 | 0.55 | 0.55 | 0.51 | 0.59 | 0.58 |
| 6e62_A_B_C | 0.55 | 0.55 | 0.64 | 0.61 | 0.88 | 0.67 | 0.65 |
| 6e63_P_H_L | 0.58 | 0.57 | 0.59 | 0.60 | 0.88 | 0.67 | 0.62 |
| 6eay_A_H_L | 0.63 | 0.98 | 0.73 | 0.64 | 0.95 | 0.66 | 0.60 |
| 6edu_D_L_M | 0.66 | 0.53 | 0.52 | 0.52 | 0.55 | 0.52 | 0.54 |
| 6edu_F_P_Q | 0.68 | 0.50 | 0.53 | 0.52 | 0.62 | 0.59 | 0.57 |
| 6elu_D_E_F | 0.50 | 0.99 | 0.77 | 0.70 | 0.98 | 0.82 | 0.69 |
| 6eti_A_F_E | 0.82 | 0.52 | 0.52 | 0.52 | 0.52 | 0.52 | 0.52 |
| 6ewb_C_G_I | 0.80 | 0.53 | 0.60 | 0.58 | 0.90 | 0.61 | 0.57 |
| 6eyo_A_H_L | 0.66 | 1.00 | 0.69 | 0.61 | 0.96 | 0.73 | 0.69 |
| 6fax_R_H_L | 0.60 | 0.99 | 0.76 | 0.74 | 0.97 | 0.72 | 0.70 |
| 6feq_A_F_E | 0.84 | 0.52 | 0.52 | 0.52 | 0.51 | 0.51 | 0.52 |
| 6fgb_A_H_L | 0.59 | 0.55 | 0.66 | 0.61 | 0.53 | 0.52 | 0.53 |
| 6fla_I_H_L | 0.50 | 0.60 | 0.66 | 0.62 | 0.61 | 0.59 | 0.65 |
| 6flb_G_H_L | 0.52 | 0.98 | 0.69 | 0.64 | 0.83 | 0.59 | 0.56 |
| 6flc_G_H_L | 0.76 | 0.60 | 0.58 | 0.57 | 0.52 | 0.62 | 0.68 |
| 6fn1_A_C_B | 0.80 | 0.54 | 0.52 | 0.51 | 0.87 | 0.60 | 0.59 |
| 6fn4_A_C_B | 0.82 | 0.51 | 0.52 | 0.52 | 0.90 | 0.60 | 0.62 |
| 6fxn_A_D_E | 0.61 | 0.96 | 0.65 | 0.65 | 0.56 | 0.56 | 0.60 |
| 6fy1_G_H_L | 0.81 | 0.55 | 0.58 | 0.61 | 0.91 | 0.76 | 0.71 |
| 6gv1_A_H_L | 0.83 | 0.53 | 0.52 | 0.53 | 0.52 | 0.60 | 0.58 |
| 6h3t_B_I_M | 0.69 | 0.99 | 0.67 | 0.72 | 0.55 | 0.55 | 0.60 |
| 6h3u_B_H_L | 0.51 | 0.99 | 0.64 | 0.60 | 0.60 | 0.56 | 0.59 |
| 6h5n_A_C_B | 0.69 | 0.58 | 0.58 | 0.58 | 0.54 | 0.57 | 0.63 |
| 6hco_B_D_C | 0.88 | 0.53 | 0.52 | 0.52 | 0.52 | 0.53 | 0.52 |
| 6hf1_A_C_B | 0.70 | 0.54 | 0.55 | 0.55 | 0.54 | 0.56 | 0.57 |
| 6i04_A_H_L | 0.80 | 0.99 | 0.83 | 0.71 | 0.85 | 0.61 | 0.57 |
| 6i8s_D_H_L | 0.81 | 1.00 | 0.73 | 0.66 | 0.94 | 0.60 | 0.60 |
| 6i9i_D_H_L | 0.55 | 0.72 | 0.70 | 0.69 | 0.81 | 0.67 | 0.68 |
| 6iea_A_H_L | 0.76 | 0.53 | 0.53 | 0.53 | 0.53 | 0.54 | 0.54 |
| 6ieb_A_H_L | 0.71 | 0.53 | 0.53 | 0.53 | 0.55 | 0.53 | 0.53 |
| 6iec_A_H_L | 0.76 | 0.55 | 0.65 | 0.59 | 0.52 | 0.61 | 0.57 |
| 6iek_A_B_C | 0.74 | 0.54 | 0.54 | 0.53 | 0.53 | 0.53 | 0.53 |
| 6ii4_A_H_L | 0.50 | 0.53 | 0.62 | 0.57 | 0.99 | 0.65 | 0.59 |
| 6ii8_C_H_L | 0.61 | 0.53 | 0.53 | 0.53 | 0.51 | 0.52 | 0.57 |
| 6ii9_C_M_N | 0.62 | 0.55 | 0.53 | 0.58 | 0.53 | 0.57 | 0.59 |
| 6iut_A_H_L | 0.65 | 1.00 | 0.70 | 0.72 | 0.53 | 0.67 | 0.61 |
| 6iuv_B_C_D | 0.64 | 0.99 | 0.62 | 0.63 | 0.50 | 0.56 | 0.57 |
| 6ivz_A_H_L | 0.60 | 0.53 | 0.63 | 0.58 | 0.52 | 0.52 | 0.53 |
| 6iw2_J_K_L | 0.66 | 0.52 | 0.54 | 0.55 | 0.53 | 0.53 | 0.57 |
| 6j5d_A_H_L | 0.79 | 0.59 | 0.59 | 0.59 | 0.67 | 0.66 | 0.62 |
| 6j5g_A_H_L | 0.75 | 0.52 | 0.52 | 0.52 | 0.51 | 0.62 | 0.60 |
| 6mar_B_M_N | 0.60 | 0.59 | 0.58 | 0.60 | 0.57 | 0.58 | 0.65 |
| 6mdt_B_D_E | 0.80 | 0.54 | 0.54 | 0.57 | 0.55 | 0.56 | 0.56 |
| 6mdt_G_H_L | 0.82 | 0.52 | 0.52 | 0.53 | 0.52 | 0.52 | 0.55 |
| 6meh_C_H_L | 0.64 | 0.97 | 0.70 | 0.70 | 0.88 | 0.68 | 0.65 |
| 6mei_C_H_L | 0.64 | 0.99 | 0.89 | 0.79 | 0.66 | 0.68 | 0.62 |
| 6mej_C_A_B | 0.58 | 0.55 | 0.55 | 0.59 | 0.54 | 0.62 | 0.58 |
| 6mej_C_H_L | 0.81 | 0.99 | 0.65 | 0.63 | 0.55 | 0.63 | 0.60 |
| 6mek_A_B_D | 0.65 | 1.00 | 0.70 | 0.69 | 0.55 | 0.62 | 0.61 |
| 6mek_C_F_E | 0.59 | 0.57 | 0.57 | 0.56 | 0.54 | 0.55 | 0.61 |
| 6mft_G_H_L | 0.94 | 0.55 | 0.52 | 0.52 | 0.57 | 0.54 | 0.62 |
| 6mhr_C_A_B | 0.86 | 0.59 | 0.58 | 0.58 | 0.60 | 0.65 | 0.61 |
| 6mi2_C_A_B | 0.57 | 0.57 | 0.57 | 0.60 | 0.57 | 0.57 | 0.59 |
| 6ml8_A_H_L | 0.76 | 0.53 | 0.59 | 0.59 | 0.89 | 0.69 | 0.64 |
| 6mlk_A_H_L | 0.68 | 0.84 | 0.61 | 0.62 | 0.95 | 0.69 | 0.64 |
| 6mlm_A_E_I | 0.68 | 0.53 | 0.56 | 0.55 | 0.53 | 0.54 | 0.54 |
| 6mto_T_H_L | 0.76 | 0.94 | 0.85 | 0.78 | 0.56 | 0.55 | 0.55 |
| 6mtq_T_H_L | 0.80 | 0.96 | 0.80 | 0.79 | 0.78 | 0.74 | 0.70 |
| 6mu6_G_H_L | 0.85 | 0.52 | 0.53 | 0.53 | 0.96 | 0.70 | 0.67 |
| 6mu7_B_D_E | 0.77 | 0.57 | 0.54 | 0.55 | 0.59 | 0.58 | 0.58 |
| 6mu7_G_H_L | 0.89 | 0.52 | 0.54 | 0.53 | 0.88 | 0.72 | 0.68 |
| 6mu8_G_H_L | 0.90 | 0.50 | 0.54 | 0.54 | 0.75 | 0.78 | 0.70 |
| 6muf_G_H_L | 0.90 | 0.53 | 0.56 | 0.54 | 0.73 | 0.56 | 0.61 |
| 6mug_G_H_L | 0.87 | 0.52 | 0.52 | 0.57 | 0.52 | 0.58 | 0.56 |
| 6mui_B_C_D | 0.76 | 0.54 | 0.55 | 0.54 | 0.52 | 0.55 | 0.54 |
| 6mw9_B_C_D | 0.85 | 0.54 | 0.53 | 0.53 | 0.53 | 0.53 | 0.53 |
| 6mwc_B_C_D | 0.75 | 0.54 | 0.53 | 0.54 | 0.54 | 0.53 | 0.55 |
| 6mwv_J_K_L | 0.76 | 0.54 | 0.54 | 0.53 | 0.58 | 0.53 | 0.55 |
| 6mwx_J_K_L | 0.80 | 0.53 | 0.53 | 0.53 | 0.53 | 0.53 | 0.53 |
| 6mwx_N_O_P | 0.98 | 0.52 | 0.52 | 0.53 | 0.53 | 0.53 | 0.53 |
| 6myy_E_G_F | 0.92 | 0.52 | 0.53 | 0.56 | 0.86 | 0.75 | 0.66 |
| 6nb3_C_H_L | 0.78 | 1.00 | 0.61 | 0.56 | 1.00 | 0.61 | 0.56 |
| 6nm6_G_H_L | 0.84 | 0.52 | 0.54 | 0.54 | 0.53 | 0.52 | 0.52 |
| 6nm6_G_U_V | 0.87 | 0.52 | 0.52 | 0.53 | 0.54 | 0.62 | 0.61 |
| 6nn3_A_H_L | 0.71 | 0.52 | 0.53 | 0.53 | 0.51 | 0.60 | 0.57 |
| 6nnf_G_H_L | 0.83 | 0.52 | 0.56 | 0.54 | 0.53 | 0.52 | 0.62 |
| 6nnf_G_U_V | 0.93 | 0.53 | 0.53 | 0.52 | 0.63 | 0.57 | 0.57 |
| 6nnj_G_U_V | 0.86 | 0.52 | 0.53 | 0.53 | 0.56 | 0.53 | 0.54 |
| 6o39_C_B_A | 0.82 | 0.98 | 0.87 | 0.78 | 0.76 | 0.70 | 0.65 |
| 6o3a_E_B_A | 0.60 | 0.98 | 0.93 | 0.93 | 0.90 | 0.75 | 0.73 |
| 6o3b_H_G_E | 0.56 | 1.00 | 0.84 | 0.85 | 0.75 | 0.72 | 0.72 |
| 6qee_A_C_B | 0.82 | 0.51 | 0.51 | 0.51 | 0.88 | 0.58 | 0.56 |
| 6qex_A_C_B | 0.88 | 0.51 | 0.51 | 0.51 | 0.93 | 0.59 | 0.56 |
| 6r2s_C_B_A | 0.52 | 0.52 | 0.52 | 0.52 | 0.53 | 0.63 | 0.58 |
| 6r8x_A_C_B | 0.58 | 1.00 | 0.91 | 0.81 | 0.96 | 0.63 | 0.62 |

^a^ The prediction is based on the original crystallized structure of antigen and the original crystallized structure of antibody.

**Supplementary Table 5. Performance of Patch model compared with popular peers on 193 testing datasets based on modelled antibody structure**

| Modelled antibody | Patch Model | | ZDOCK_T1 | ZDOCK_T5 | ZDOCK_T10 | ClusPro_T1 | ClusPro_T5 | ClusPro_T10 |
| --- | --- | --- | --- | --- | --- | --- | --- | --- |
| 5bjz_B_D_H | 0.71 | 0.53 | | 0.60 | 0.61 | 0.60 | 0.57 | 0.56 |
| 5bk1_A_H_L | 0.55 | 0.54 | | 0.56 | 0.60 | 0.51 | 0.62 | 0.58 |
| 5bk2_A_H_L | 0.59 | 0.60 | | 0.56 | 0.57 | 0.53 | 0.53 | 0.53 |
| 5otj_C_H_L | 0.74 | 0.57 | | 0.64 | 0.70 | 0.55 | 0.64 | 0.62 |
| 5wdu_G_D_E | 0.82 | 0.53 | | 0.52 | 0.53 | 0.53 | 0.59 | 0.61 |
| 5whk_A_H_L | 0.80 | 0.53 | | 0.54 | 0.58 | 0.91 | 0.68 | 0.66 |
| 5wi9_A_H_L | 0.88 | 0.53 | | 0.54 | 0.54 | 0.51 | 0.54 | 0.53 |
| 5wob_G_U_V | 0.85 | 0.51 | | 0.51 | 0.51 | 0.51 | 0.51 | 0.51 |
| 5xxy_A_H_L | 0.71 | 0.88 | | 0.66 | 0.60 | 0.63 | 0.62 | 0.65 |
| 5y11_C_A_B | 0.65 | 0.53 | | 0.78 | 0.65 | 0.53 | 0.60 | 0.62 |
| 5y2l_B_I_J | 0.77 | 0.56 | | 0.69 | 0.62 | 0.53 | 0.53 | 0.54 |
| 5y9j_A_H_L | 0.74 | 0.58 | | 0.56 | 0.56 | 0.54 | 0.57 | 0.57 |
| 5yoy_K_P_M | 0.82 | 0.50 | | 0.55 | 0.56 | 0.64 | 0.62 | 0.59 |
| 5ywy_A_H_L | 0.90 | 0.50 | | 0.52 | 0.52 | 0.53 | 0.54 | 0.58 |
| 5yy5_A_H_L | 0.75 | 0.53 | | 0.53 | 0.53 | 0.52 | 0.56 | 0.57 |
| 5zxv_A_H_L | 0.83 | 0.67 | | 0.63 | 0.64 | 0.54 | 0.56 | 0.61 |
| 6a0z_A_H_L | 0.63 | 0.52 | | 0.53 | 0.53 | 0.56 | 0.54 | 0.55 |
| 6a3w_C_A_B | 0.68 | 0.60 | | 0.59 | 0.58 | 0.56 | 0.58 | 0.59 |
| 6a4k_B_I_M | 0.71 | 0.56 | | 0.55 | 0.55 | 0.55 | 0.59 | 0.58 |
| 6a67_A_H_L | 0.74 | 0.82 | | 0.75 | 0.67 | 0.99 | 0.71 | 0.64 |
| 6a78_A_H_L | 0.64 | 0.83 | | 0.72 | 0.69 | 0.65 | 0.70 | 0.71 |
| 6al5_A_H_L | 0.52 | 0.60 | | 0.55 | 0.55 | 0.55 | 0.57 | 0.56 |
| 6aod_C_B_A | 0.53 | 0.54 | | 0.66 | 0.64 | 0.68 | 0.65 | 0.64 |
| 6apb_C_H_L | 0.50 | 0.52 | | 0.53 | 0.53 | 0.52 | 0.52 | 0.53 |
| 6apd_C_F_G | 0.88 | 0.51 | | 0.52 | 0.52 | 0.51 | 0.51 | 0.51 |
| 6apd_C_J_L | 0.85 | 0.53 | | 0.52 | 0.52 | 0.52 | 0.59 | 0.57 |
| 6aq7_A_H_L | 0.52 | 0.73 | | 0.65 | 0.63 | 0.75 | 0.72 | 0.72 |
| 6aru_A_C_B | 0.96 | 0.92 | | 0.60 | 0.56 | 0.88 | 0.62 | 0.60 |
| 6att_A_H_L | 0.75 | 0.52 | | 0.52 | 0.52 | 0.52 | 0.52 | 0.54 |
| 6ayz_A_B_D | 0.70 | 0.59 | | 0.65 | 0.62 | 0.57 | 0.59 | 0.58 |
| 6az2_B_A_F | 0.55 | 0.59 | | 0.56 | 0.56 | 0.53 | 0.55 | 0.55 |
| 6azz_A_C_B | 0.78 | 0.62 | | 0.57 | 0.62 | 0.59 | 0.57 | 0.59 |
| 6b08_A_C_B | 0.74 | 0.56 | | 0.64 | 0.60 | 0.99 | 0.63 | 0.59 |
| 6b0a_A_H_L | 0.58 | 0.60 | | 0.69 | 0.69 | 0.64 | 0.72 | 0.65 |
| 6b0e_E_B_A | 0.53 | 0.90 | | 0.70 | 0.68 | 0.91 | 0.68 | 0.65 |
| 6b0g_E_D_C | 0.50 | 0.54 | | 0.71 | 0.70 | 0.86 | 0.67 | 0.64 |
| 6b0h_I_D_C | 0.69 | 0.57 | | 0.66 | 0.63 | 0.50 | 0.58 | 0.56 |
| 6b0n_G_D_E | 0.84 | 0.51 | | 0.51 | 0.51 | 0.52 | 0.52 | 0.55 |
| 6b0n_G_H_L | 0.92 | 0.52 | | 0.52 | 0.52 | 0.53 | 0.53 | 0.53 |
| 6b0s_C_H_L | 0.76 | 0.64 | | 0.64 | 0.64 | 0.95 | 0.65 | 0.63 |
| 6b3s_A_H_L | 0.75 | 0.84 | | 0.67 | 0.64 | 0.58 | 0.61 | 0.60 |
| 6b70_B_E_F | 0.82 | 0.51 | | 0.52 | 0.52 | 0.51 | 0.51 | 0.51 |
| 6b7z_B_E_F | 0.92 | 0.51 | | 0.51 | 0.51 | 0.51 | 0.51 | 0.51 |
| 6b9j_X_H_L | 0.68 | 0.56 | | 0.55 | 0.54 | 0.76 | 0.69 | 0.68 |
| 6bae_E_B_A | 0.91 | 0.89 | | 0.71 | 0.71 | 0.60 | 0.61 | 0.61 |
| 6bah_C_B_A | 0.88 | 0.73 | | 0.66 | 0.63 | 0.90 | 0.63 | 0.67 |
| 6bck_G_H_L | 0.93 | 0.54 | | 0.53 | 0.53 | 0.71 | 0.61 | 0.60 |
| 6bdz_A_H_L | 0.85 | 0.52 | | 0.52 | 0.52 | 0.52 | 0.52 | 0.53 |
| 6bf7_B_E_F | 0.83 | 0.51 | | 0.51 | 0.51 | 0.51 | 0.52 | 0.54 |
| 6bf9_A_C_D | 0.90 | 0.51 | | 0.51 | 0.51 | 0.51 | 0.51 | 0.53 |
| 6bfq_K_H_L | 0.69 | 0.88 | | 0.71 | 0.66 | 0.86 | 0.64 | 0.66 |
| 6bfs_C_H_L | 0.84 | 0.89 | | 0.91 | 0.90 | 0.60 | 0.61 | 0.68 |
| 6bft_C_H_L | 0.90 | 0.62 | | 0.61 | 0.60 | 0.96 | 0.73 | 0.69 |
| 6bft_G_A_B | 0.88 | 0.60 | | 0.61 | 0.63 | 0.95 | 0.72 | 0.65 |
| 6bgt_C_B_A | 0.85 | 0.63 | | 0.61 | 0.62 | 0.52 | 0.52 | 0.52 |
| 6bit_G_J_L | 0.89 | 0.81 | | 0.61 | 0.64 | 0.85 | 0.72 | 0.68 |
| 6bkb_E_H_L | 0.80 | 0.55 | | 0.63 | 0.67 | 0.56 | 0.69 | 0.70 |
| 6bkc_E_H_L | 0.62 | 0.66 | | 0.74 | 0.70 | 0.85 | 0.78 | 0.71 |
| 6bkd_E_H_L | 0.77 | 0.60 | | 0.63 | 0.67 | 0.79 | 0.66 | 0.69 |
| 6bp2_A_H_L | 0.67 | 0.52 | | 0.61 | 0.59 | 0.57 | 0.56 | 0.57 |
| 6bpa_A_B_C | 0.63 | 0.53 | | 0.53 | 0.53 | 0.52 | 0.52 | 0.53 |
| 6bpc_A_B_C | 0.76 | 0.55 | | 0.53 | 0.53 | 0.52 | 0.53 | 0.58 |
| 6bpe_D_E_F | 0.59 | 0.54 | | 0.54 | 0.54 | 0.53 | 0.55 | 0.55 |
| 6c5v_A_H_L | 0.70 | 0.85 | | 0.65 | 0.68 | 0.51 | 0.52 | 0.56 |
| 6c6y_R_H_L | 0.81 | 0.93 | | 0.69 | 0.70 | 0.55 | 0.58 | 0.59 |
| 6c6z_B_H_L | 0.89 | 0.55 | | 0.65 | 0.63 | 0.90 | 0.71 | 0.67 |
| 6c9u_A_H_L | 0.79 | 0.81 | | 0.67 | 0.65 | 0.63 | 0.57 | 0.55 |
| 6cbv_B_H_L | 0.59 | 0.58 | | 0.60 | 0.60 | 0.58 | 0.58 | 0.59 |
| 6cde_C_M_N | 0.81 | 0.52 | | 0.52 | 0.52 | 0.53 | 0.53 | 0.53 |
| 6cde_C_Q_R | 0.92 | 0.53 | | 0.53 | 0.54 | 0.52 | 0.64 | 0.63 |
| 6cdi_C_Q_R | 0.90 | 0.54 | | 0.53 | 0.54 | 0.52 | 0.52 | 0.58 |
| 6ce0_B_D_E | 0.50 | 0.59 | | 0.56 | 0.56 | 0.61 | 0.58 | 0.58 |
| 6cf2_F_A_B | 0.54 | 0.64 | | 0.64 | 0.65 | 0.67 | 0.62 | 0.61 |
| 6ch7_B_D_E | 0.76 | 0.75 | | 0.60 | 0.58 | 0.53 | 0.56 | 0.56 |
| 6chb_F_M_N | 0.84 | 0.52 | | 0.53 | 0.52 | 0.51 | 0.52 | 0.52 |
| 6chb_G_D_E | 0.78 | 0.52 | | 0.52 | 0.52 | 0.51 | 0.55 | 0.55 |
| 6chb_G_J_K | 0.75 | 0.52 | | 0.53 | 0.52 | 0.53 | 0.63 | 0.60 |
| 6chb_H_O_P | 0.69 | 0.51 | | 0.51 | 0.51 | 0.51 | 0.51 | 0.51 |
| 6ck9_G_H_L | 0.88 | 0.52 | | 0.53 | 0.53 | 0.52 | 0.59 | 0.56 |
| 6cm3_A_R_S | 0.89 | 0.61 | | 0.59 | 0.58 | 0.56 | 0.63 | 0.62 |
| 6cm3_E_O_N | 0.96 | 0.52 | | 0.67 | 0.60 | 0.52 | 0.60 | 0.58 |
| 6cmg_A_C_B | 0.56 | 0.53 | | 0.52 | 0.52 | 0.52 | 0.57 | 0.57 |
| 6cmi_B_D_C | 0.51 | 0.53 | | 0.52 | 0.52 | 0.60 | 0.59 | 0.56 |
| 6cmo_A_H_L | 0.55 | 0.51 | | 0.52 | 0.53 | 0.52 | 0.53 | 0.53 |
| 6cnv_B_H_L | 0.62 | 0.73 | | 0.59 | 0.58 | 0.55 | 0.56 | 0.55 |
| 6crq_A_D_E | 0.93 | 0.53 | | 0.53 | 0.52 | 0.76 | 0.60 | 0.61 |
| 6cse_M_H_L | 0.76 | 0.52 | | 0.53 | 0.52 | 0.52 | 0.52 | 0.52 |
| 6csf_M_H_L | 0.78 | 0.53 | | 0.53 | 0.52 | 0.52 | 0.52 | 0.52 |
| 6cue_C_Q_R | 0.92 | 0.52 | | 0.52 | 0.52 | 0.94 | 0.61 | 0.59 |
| 6cuf_d_m_n | 0.91 | 0.53 | | 0.52 | 0.52 | 0.52 | 0.59 | 0.56 |
| 6cuf_d_q_r | 0.90 | 0.53 | | 0.53 | 0.54 | 0.52 | 0.58 | 0.60 |
| 6cw2_D_A_B | 0.57 | 0.55 | | 0.63 | 0.63 | 0.54 | 0.63 | 0.67 |
| 6cw3_H_A_B | 0.50 | 0.51 | | 0.51 | 0.51 | 0.53 | 0.52 | 0.52 |
| 6cwt_F_C_D | 0.61 | 0.56 | | 0.56 | 0.56 | 0.58 | 0.56 | 0.57 |
| 6cxy_C_H_L | 0.72 | 0.51 | | 0.53 | 0.53 | 0.59 | 0.60 | 0.56 |
| 6cyf_A_D_C | 0.63 | 0.60 | | 0.58 | 0.57 | 0.75 | 0.75 | 0.67 |
| 6d0u_G_H_I | 0.94 | 0.51 | | 0.53 | 0.53 | 0.53 | 0.54 | 0.55 |
| 6d2p_C_H_L | 0.86 | 0.52 | | 0.58 | 0.56 | 0.60 | 0.64 | 0.64 |
| 6d6t_B_J_I | 0.60 | 0.54 | | 0.53 | 0.53 | 0.56 | 0.53 | 0.58 |
| 6d6u_B_J_I | 0.62 | 0.53 | | 0.53 | 0.53 | 0.55 | 0.53 | 0.54 |
| 6ddm_C_B_A | 0.70 | 0.56 | | 0.59 | 0.57 | 0.67 | 0.67 | 0.64 |
| 6ddr_C_B_A | 0.63 | 0.62 | | 0.61 | 0.63 | 0.61 | 0.61 | 0.61 |
| 6ddv_C_B_A | 0.78 | 0.56 | | 0.78 | 0.73 | 0.97 | 0.65 | 0.63 |
| 6de7_G_H_L | 0.89 | 0.53 | | 0.53 | 0.53 | 0.61 | 0.54 | 0.55 |
| 6dfi_E_H_L | 0.79 | 0.59 | | 0.59 | 0.61 | 0.59 | 0.65 | 0.64 |
| 6dfj_E_H_L | 0.78 | 0.61 | | 0.60 | 0.64 | 0.58 | 0.66 | 0.61 |
| 6did_G_K_E | 0.52 | 0.53 | | 0.52 | 0.52 | 0.52 | 0.58 | 0.56 |
| 6e3h_B_H_L | 0.88 | 0.57 | | 0.56 | 0.56 | 0.54 | 0.55 | 0.55 |
| 6e62_A_B_C | 0.59 | 0.52 | | 0.59 | 0.60 | 0.53 | 0.65 | 0.61 |
| 6e63_P_H_L | 0.65 | 0.60 | | 0.60 | 0.58 | 0.60 | 0.56 | 0.58 |
| 6eay_A_H_L | 0.58 | 0.51 | | 0.55 | 0.56 | 0.59 | 0.58 | 0.59 |
| 6edu_D_L_M | 0.64 | 0.52 | | 0.52 | 0.52 | 0.52 | 0.56 | 0.57 |
| 6edu_F_P_Q | 0.64 | 0.51 | | 0.52 | 0.52 | 0.54 | 0.54 | 0.54 |
| 6elu_D_E_F | 0.66 | 0.76 | | 0.60 | 0.57 | 0.98 | 0.75 | 0.67 |
| 6eti_A_F_E | 0.80 | 0.52 | | 0.52 | 0.52 | 0.52 | 0.52 | 0.52 |
| 6ewb_C_G_I | 0.75 | 0.53 | | 0.53 | 0.54 | 0.53 | 0.53 | 0.53 |
| 6eyo_A_H_L | 0.69 | 0.53 | | 0.53 | 0.56 | 0.86 | 0.62 | 0.60 |
| 6fax_R_H_L | 0.58 | 0.59 | | 0.58 | 0.57 | 0.59 | 0.65 | 0.67 |
| 6feq_A_F_E | 0.81 | 0.52 | | 0.52 | 0.52 | 0.51 | 0.51 | 0.52 |
| 6fgb_A_H_L | 0.61 | 0.54 | | 0.54 | 0.54 | 0.53 | 0.55 | 0.56 |
| 6fla_I_H_L | 0.54 | 0.54 | | 0.58 | 0.59 | 0.62 | 0.58 | 0.57 |
| 6flb_G_H_L | 0.51 | 0.64 | | 0.61 | 0.60 | 0.60 | 0.64 | 0.61 |
| 6flc_G_H_L | 0.76 | 0.58 | | 0.58 | 0.59 | 0.66 | 0.58 | 0.65 |
| 6fn1_A_C_B | 0.80 | 0.51 | | 0.51 | 0.51 | 0.51 | 0.51 | 0.51 |
| 6fn4_A_C_B | 0.80 | 0.51 | | 0.51 | 0.51 | 0.51 | 0.51 | 0.51 |
| 6fxn_A_D_E | 0.72 | 0.56 | | 0.56 | 0.57 | 0.54 | 0.55 | 0.55 |
| 6fy1_G_H_L | 0.75 | 0.71 | | 0.63 | 0.61 | 0.85 | 0.69 | 0.69 |
| 6gv1_A_H_L | 0.80 | 0.54 | | 0.52 | 0.53 | 0.52 | 0.52 | 0.52 |
| 6h3t_B_I_M | 0.67 | 0.56 | | 0.53 | 0.54 | 0.54 | 0.55 | 0.54 |
| 6h3u_B_H_L | 0.64 | 0.63 | | 0.58 | 0.57 | 0.53 | 0.55 | 0.54 |
| 6h5n_A_C_B | 0.61 | 0.60 | | 0.67 | 0.66 | 0.62 | 0.61 | 0.61 |
| 6hco_B_D_C | 0.87 | 0.52 | | 0.52 | 0.52 | 0.52 | 0.52 | 0.52 |
| 6hf1_A_C_B | 0.63 | 0.54 | | 0.54 | 0.55 | 0.70 | 0.58 | 0.57 |
| 6i04_A_H_L | 0.75 | 0.83 | | 0.58 | 0.58 | 0.52 | 0.54 | 0.57 |
| 6i8s_D_H_L | 0.72 | 0.54 | | 0.57 | 0.60 | 0.53 | 0.60 | 0.57 |
| 6i9i_D_H_L | 0.64 | 0.57 | | 0.61 | 0.61 | 0.90 | 0.71 | 0.63 |
| 6iea_A_H_L | 0.80 | 0.50 | | 0.53 | 0.53 | 0.55 | 0.59 | 0.56 |
| 6ieb_A_H_L | 0.75 | 0.54 | | 0.54 | 0.54 | 0.55 | 0.54 | 0.56 |
| 6iec_A_H_L | 0.83 | 0.55 | | 0.54 | 0.54 | 0.53 | 0.52 | 0.53 |
| 6iek_A_B_C | 0.74 | 0.55 | | 0.52 | 0.53 | 0.52 | 0.52 | 0.53 |
| 6ii4_A_H_L | 0.56 | 0.54 | | 0.53 | 0.53 | 0.52 | 0.61 | 0.57 |
| 6ii8_C_H_L | 0.56 | 0.54 | | 0.53 | 0.53 | 0.53 | 0.53 | 0.53 |
| 6ii9_C_M_N | 0.54 | 0.54 | | 0.53 | 0.53 | 0.53 | 0.62 | 0.57 |
| 6iut_A_H_L | 0.67 | 0.54 | | 0.54 | 0.58 | 0.53 | 0.55 | 0.56 |
| 6iuv_B_C_D | 0.57 | 0.52 | | 0.54 | 0.55 | 0.54 | 0.54 | 0.54 |
| 6ivz_A_H_L | 0.62 | 0.54 | | 0.55 | 0.54 | 0.53 | 0.54 | 0.54 |
| 6iw2_J_K_L | 0.66 | 0.58 | | 0.55 | 0.55 | 0.54 | 0.53 | 0.53 |
| 6j5d_A_H_L | 0.65 | 0.63 | | 0.65 | 0.63 | 0.56 | 0.59 | 0.58 |
| 6j5g_A_H_L | 0.75 | 0.52 | | 0.52 | 0.52 | 0.51 | 0.52 | 0.55 |
| 6mar_B_M_N | 0.58 | 0.64 | | 0.63 | 0.61 | 0.55 | 0.57 | 0.58 |
| 6mdt_B_D_E | 0.60 | 0.57 | | 0.58 | 0.57 | 0.52 | 0.54 | 0.55 |
| 6mdt_G_H_L | 0.87 | 0.52 | | 0.52 | 0.52 | 0.52 | 0.52 | 0.52 |
| 6meh_C_H_L | 0.57 | 0.85 | | 0.91 | 0.79 | 0.90 | 0.69 | 0.68 |
| 6mei_C_H_L | 0.59 | 0.94 | | 0.75 | 0.70 | 0.55 | 0.69 | 0.67 |
| 6mej_C_A_B | 0.52 | 0.55 | | 0.56 | 0.56 | 0.56 | 0.60 | 0.57 |
| 6mej_C_H_L | 0.79 | 0.95 | | 0.66 | 0.61 | 0.58 | 0.63 | 0.65 |
| 6mek_A_B_D | 0.65 | 0.68 | | 0.68 | 0.66 | 0.52 | 0.60 | 0.62 |
| 6mek_C_F_E | 0.65 | 0.57 | | 0.56 | 0.57 | 0.55 | 0.55 | 0.56 |
| 6mft_G_H_L | 0.94 | 0.52 | | 0.53 | 0.53 | 0.55 | 0.53 | 0.61 |
| 6mhr_C_A_B | 0.86 | 0.57 | | 0.56 | 0.56 | 0.58 | 0.57 | 0.57 |
| 6mi2_C_A_B | 0.57 | 0.59 | | 0.57 | 0.60 | 0.57 | 0.58 | 0.58 |
| 6ml8_A_H_L | 0.71 | 0.58 | | 0.57 | 0.57 | 0.51 | 0.53 | 0.58 |
| 6mlk_A_H_L | 0.68 | 0.54 | | 0.72 | 0.70 | 0.89 | 0.66 | 0.66 |
| 6mlm_A_E_I | 0.67 | 0.53 | | 0.53 | 0.56 | 0.53 | 0.52 | 0.56 |
| 6mto_T_H_L | 0.78 | 0.83 | | 0.82 | 0.82 | 0.52 | 0.54 | 0.54 |
| 6mtq_T_H_L | 0.79 | 0.73 | | 0.66 | 0.67 | 0.86 | 0.73 | 0.69 |
| 6mu6_G_H_L | 0.81 | 0.60 | | 0.56 | 0.54 | 0.96 | 0.67 | 0.65 |
| 6mu7_B_D_E | 0.66 | 0.54 | | 0.54 | 0.55 | 0.59 | 0.59 | 0.57 |
| 6mu7_G_H_L | 0.81 | 0.53 | | 0.54 | 0.54 | 0.76 | 0.71 | 0.69 |
| 6mu8_G_H_L | 0.87 | 0.52 | | 0.54 | 0.54 | 0.78 | 0.75 | 0.66 |
| 6muf_G_H_L | 0.84 | 0.52 | | 0.52 | 0.52 | 0.52 | 0.52 | 0.57 |
| 6mug_G_H_L | 0.82 | 0.52 | | 0.52 | 0.52 | 0.52 | 0.57 | 0.55 |
| 6mui_B_C_D | 0.74 | 0.52 | | 0.60 | 0.57 | 0.54 | 0.57 | 0.56 |
| 6mw9_B_C_D | 0.82 | 0.52 | | 0.54 | 0.54 | 0.54 | 0.53 | 0.53 |
| 6mwc_B_C_D | 0.76 | 0.52 | | 0.56 | 0.54 | 0.54 | 0.55 | 0.54 |
| 6mwv_J_K_L | 0.75 | 0.55 | | 0.56 | 0.55 | 0.54 | 0.57 | 0.55 |
| 6mwx_J_K_L | 0.78 | 0.54 | | 0.55 | 0.54 | 0.55 | 0.54 | 0.53 |
| 6mwx_N_O_P | 0.96 | 0.53 | | 0.53 | 0.53 | 0.54 | 0.54 | 0.53 |
| 6myy_E_G_F | 0.91 | 0.52 | | 0.52 | 0.52 | 0.52 | 0.65 | 0.67 |
| 6nb3_C_H_L | 0.63 | 0.51 | | 0.51 | 0.51 | 0.51 | 0.51 | 0.51 |
| 6nm6_G_H_L | 0.83 | 0.52 | | 0.53 | 0.53 | 0.53 | 0.57 | 0.54 |
| 6nm6_G_U_V | 0.90 | 0.53 | | 0.52 | 0.53 | 0.56 | 0.59 | 0.57 |
| 6nn3_A_H_L | 0.69 | 0.52 | | 0.52 | 0.52 | 0.53 | 0.53 | 0.60 |
| 6nnf_G_H_L | 0.78 | 0.52 | | 0.52 | 0.52 | 0.53 | 0.53 | 0.52 |
| 6nnf_G_U_V | 0.93 | 0.54 | | 0.53 | 0.53 | 0.53 | 0.61 | 0.63 |
| 6nnj_G_U_V | 0.87 | 0.52 | | 0.52 | 0.52 | 0.52 | 0.52 | 0.52 |
| 6o39_C_B_A | 0.68 | 0.85 | | 0.78 | 0.75 | 0.85 | 0.71 | 0.64 |
| 6o3a_E_B_A | 0.50 | 0.86 | | 0.88 | 0.83 | 0.78 | 0.60 | 0.63 |
| 6o3b_H_G_E | 0.55 | 0.56 | | 0.54 | 0.55 | 0.61 | 0.56 | 0.57 |
| 6qee_A_C_B | 0.80 | 0.51 | | 0.51 | 0.51 | 0.68 | 0.54 | 0.55 |
| 6qex_A_C_B | 0.87 | 0.51 | | 0.51 | 0.51 | 0.51 | 0.54 | 0.57 |
| 6r2s_C_B_A | 0.52 | 0.54 | | 0.54 | 0.53 | 0.53 | 0.53 | 0.53 |
| 6r8x_A_C_B | 0.56 | 0.56 | | 0.67 | 0.68 | 0.53 | 0.58 | 0.61 |

^a^ The prediction is based on the original crystallized structure of antigen and the modelled structure of antibody.

**Supplementary Table 6. Performance of SEPPA-mAb on 31 independent testing dataset of SARS-CoV-2 spike protein**

| PDB id | antigen chain | antibody chain | ACC | FPR | epitope overlapping score^a^ |
| --- | --- | --- | --- | --- | --- |
| 7JMP | A | HL | 0.661 | 0.351 | 0.818 |
| 7K45 | B | HL | 0.763 | 0.24 | 0.818 |
| 7CWS | Q | LC | 0.936 | 0.062 | 0.727 |
| 7KZB | C | AB | 0.724 | 0.272 | 0.667 |
| 7CH5 | R | HL | 0.623 | 0.38 | 0.64 |
| 7CH4 | R | HL | 0.681 | 0.311 | 0.63 |
| 7L2C | A | HL | 0.878 | 0.107 | 0.625 |
| 6XKQ | A | HL | 0.839 | 0.13 | 0.611 |
| 7LOP | Z | XY | 0.67 | 0.322 | 0.588 |
| 7BEO | X | CD | 0.681 | 0.312 | 0.583 |
| 7KN6 | A | HL | 0.604 | 0.392 | 0.571 |
| 7DPM | C | AB | 0.75 | 0.233 | 0.562 |
| 7CHC | R | AB | 0.77 | 0.215 | 0.545 |
| 7BZ5 | A | HL | 0.768 | 0.189 | 0.533 |
| 7K90 | B | HL | 0.911 | 0.084 | 0.5 |
| 6XE1 | E | HL | 0.662 | 0.314 | 0.455 |
| 7C01 | A | HL | 0.703 | 0.256 | 0.444 |
| 7BEN | E | AB | 0.621 | 0.36 | 0.438 |
| 7BYR | B | HL | 0.964 | 0.028 | 0.429 |
| 7NEG | E | HL | 0.705 | 0.248 | 0.423 |
| 7A5R | A | HL | 0.739 | 0.237 | 0.421 |
| 6XCM | C | HL | 0.934 | 0.055 | 0.368 |
| 7K8V | A | HL | 0.955 | 0.036 | 0.357 |
| 7L3N | B | DE | 0.937 | 0.051 | 0.235 |
| 6ZFO | A | BC | 0.546 | 0.413 | 0.227 |
| 7JVA | A | HL | 0.632 | 0.331 | 0.2 |
| 7CAH | A | ED | 0.777 | 0.155 | 0.167 |
| 7JMW | A | HL | 0.641 | 0.291 | 0.05 |
| 6WPT | A | DE | 0.965 | 0.023 | 0 |
| 7BEL | R | AB | 0.624 | 0.297 | 0 |
| 7KZB | C | HL | 0.677 | 0.244 | 0 |

^a^ The epitope overlapping score representing the overlapped percentage of residues between real epitope and predicted epitope.

**Supplementary Table 7. RMSD between crystalized structures and modelled structures**

| Training dataset^a^ | | | | | | | | | |
| --- | --- | --- | --- | --- | --- | --- | --- | --- | --- |
| 1a14_HL | 0.48 | 2yss_BA | 0.01 | 4d9r_HL | 0.29 | 4rx4_HL | 0.47 | 5jq6_HL | 0.02 |
| 1a2y_BA | 0.35 | 2zch_HL | 0.93 | 4dag_HL | 0.74 | 4s1q_HL | 0.03 | 5jw3_HL | 0.35 |
| 1adq_HL | 0.72 | 2zck_HL | 0.98 | 4dgi_HL | 0.22 | 4s1r_HL | 0.63 | 5jxe_DC | 0.35 |
| 1afv_HL | 0.92 | 2zcl_HL | 0.94 | 4dke_HL | 1.19 | 4s1s_HL | 0.42 | 5jz7_HL | 0.79 |
| 1ahw_BA | 1.01 | 2zjs_HL | 0.63 | 4dkf_HL | 0.88 | 4tnv_Wf | 0.73 | 5k59_EF | 0.88 |
| 1ar1_CD | 0.43 | 3a67_HL | 0.13 | 4dn4_HL | 0.79 | 4tsa_HL | 0.04 | 5k9k_HL | 0.50 |
| 1bvk_BA | 0.57 | 3a6b_HL | 0.11 | 4dqo_HL | 0.60 | 4tsb_HL | 0.03 | 5k9o_HL | 1.25 |
| 1cz8_HL | 0.24 | 3a6c_HL | 0.02 | 4dtg_HL | 0.02 | 4tsc_HL | 0.02 | 5kaq_HL | 0.68 |
| 1dee_DC | 0.42 | 3ab0_BC | 0.83 | 4dvr_HL | 0.02 | 4u0r_BC | 0.21 | 5kel_CD | 1.00 |
| 1dqj_BA | 0.16 | 3b2u_HL | 0.66 | 4dw2_HL | 0.95 | 4u1g_BC | 0.62 | 5kel_QU | 1.20 |
| 1e6j_HL | 0.93 | 3b9k_HL | 0.77 | 4edw_HL | 0.03 | 4u6h_AB | 0.64 | 5kem_BC | 3.04 |
| 1egj_HL | 0.55 | 3bdy_HL | 1.02 | 4edx_HL | 0.03 | 4u6v_KM | 0.75 | 5kem_DE | 1.66 |
| 1eo8_HL | 1.02 | 3be1_HL | 0.84 | 4etq_HL | 0.04 | 4uao_CB | 0.25 | 5ken_CD | 1.45 |
| 1fbi_HL | 1.13 | 3bgf_HL | 0.74 | 4f15_KL | 0.00 | 4ut6_IM | 0.89 | 5ken_QP | 1.39 |
| 1fdl_HL | 0.04 | 3bn9_DC | 0.75 | 4f2m_AB | 0.93 | 4ut9_JN | 0.90 | 5kjr_HL | 0.58 |
| 1fe8_HL | 0.35 | 3bt2_HL | 0.24 | 4f3f_BA | 0.26 | 4uta_HL | 0.41 | 5kqv_PQ | 0.50 |
| 1fj1_BA | 0.49 | 3c09_CB | 0.75 | 4ffv_HL | 0.02 | 4utb_HL | 0.32 | 5kvd_HL | 0.02 |
| 1fns_HL | 0.03 | 3c09_HL | 0.66 | 4ffw_DC | 0.37 | 4uu9_HL | 0.28 | 5kve_HL | 0.02 |
| 1fsk_IH | 0.69 | 3csy_AB | 0.59 | 4ffy_HL | 0.01 | 4v1d_AB | 0.52 | 5kvf_HL | 0.02 |
| 1g7h_BA | 0.24 | 3csy_EF | 0.56 | 4fp8_HL | 0.39 | 4wff_ED | 0.10 | 5kvg_HL | 0.02 |
| 1g7i_BA | 0.01 | 3cxh_JK | 0.40 | 4fqj_HL | 0.02 | 4wv1_BA | 0.09 | 5kw9_HL | 0.03 |
| 1g7j_BA | 0.14 | 3d85_BA | 0.01 | 4fqk_EF | 0.17 | 4xak_HL | 0.43 | 5kzc_HL | 0.58 |
| 1g7l_BA | 0.15 | 3d9a_HL | 0.35 | 4fqr_ab | 0.13 | 4xmn_HL | 0.67 | 5l0q_CB | 0.24 |
| 1g7m_BA | 0.24 | 3dvg_BA | 0.60 | 4fqv_JN | 0.48 | 4xmp_HL | 0.03 | 5l6y_HL | 0.03 |
| 1gc1_HL | 0.05 | 3dvn_BA | 0.58 | 4fqy_HL | 0.47 | 4xnm_HL | 0.18 | 5lcv_HL | 0.35 |
| 1h0d_BA | 0.07 | 3dvn_HL | 0.64 | 4g3y_HL | 0.38 | 4xnq_BA | 0.27 | 5lqb_HL | 0.03 |
| 1hez_BA | 1.09 | 3eff_BA | 0.77 | 4g6j_HL | 0.43 | 4xnx_HL | 0.56 | 5lsp_HL | 0.75 |
| 1hez_DC | 0.38 | 3ehb_CD | 0.24 | 4g6m_HL | 0.26 | 4xny_HL | 0.03 | 5lwy_HL | 0.02 |
| 1i9r_HL | 0.79 | 3eo1_HG | 0.65 | 4g7v_HL | 1.07 | 4xnz_EF | 0.70 | 5lxg_HL | 0.39 |
| 1ic4_HL | 0.32 | 3eoa_HL | 0.72 | 4g7y_HL | 0.96 | 4xnz_HL | 0.91 | 5mes_HL | 0.02 |
| 1ic7_HL | 0.28 | 3f7v_AB | 0.28 | 4g80_GH | 0.77 | 4xp1_HL | 0.56 | 5mev_HL | 0.39 |
| 1iqd_BA | 0.11 | 3fb5_AB | 0.30 | 4gms_HL | 0.43 | 4xp4_HL | 0.56 | 5mhr_PO | 0.48 |
| 1j1o_HL | 0.18 | 3fb8_AB | 0.19 | 4gxu_MN | 0.38 | 4xp5_HL | 0.67 | 5mi0_BC | 0.03 |
| 1j1p_HL | 0.14 | 3g04_BA | 0.43 | 4h88_HL | 0.10 | 4xp6_HL | 0.61 | 5mo9_HL | 0.68 |
| 1j1x_HL | 0.13 | 3g6d_HL | 0.40 | 4h8w_HL | 0.39 | 4xpa_HL | 0.56 | 5mvz_HL | 0.28 |
| 1jhl_HL | 0.01 | 3g6j_HG | 0.53 | 4hc1_HL | 0.45 | 4xph_HL | 0.58 | 5n09_HL | 0.38 |
| 1jps_HL | 0.03 | 3gbm_HL | 0.36 | 4hcr_HL | 0.27 | 4xrc_HL | 0.20 | 5n0a_HL | 0.26 |
| 1jrh_HL | 0.72 | 3gbn_HL | 0.02 | 4hf5_HL | 0.81 | 4xtr_EF | 0.49 | 5n7w_HL | 0.03 |
| 1kb5_HL | 0.05 | 3gi8_HL | 0.34 | 4hfu_HL | 1.11 | 4xvs_HL | 0.43 | 5ngv_HL | 0.24 |
| 1ken_TU | 0.78 | 3gi9_HL | 0.02 | 4hg4_PQ | 0.83 | 4xvt_HL | 0.04 | 5nh3_HL | 0.01 |
| 1kip_BA | 0.25 | 3grw_HL | 0.71 | 4hj0_CD | 0.98 | 4xwg_HL | 0.49 | 5nhr_HL | 0.34 |
| 1kir_BA | 0.25 | 3h42_HL | 0.02 | 4hjg_BA | 0.05 | 4xwo_OP | 0.47 | 5nj6_HL | 0.94 |
| 1kyo_JK | 0.34 | 3hb3_CD | 0.03 | 4hkx_AB | 0.00 | 4xwo_QR | 0.51 | 5njg_EF | 0.74 |
| 1lk3_IM | 0.02 | 3hfm_HL | 0.73 | 4hkz_BA | 0.53 | 4xx1_HL | 0.54 | 5nmv_HL | 0.03 |
| 1mhp_HL | 0.60 | 3hi1_HL | 0.84 | 4hlz_IJ | 0.81 | 4xzu_EF | 0.35 | 5nuz_HL | 0.14 |
| 1mlc_BA | 0.42 | 3hi6_HL | 0.30 | 4ht1_HL | 0.04 | 4y5v_AB | 0.67 | 5o14_HL | 0.03 |
| 1n6q_HL | 0.66 | 3hmx_HL | 0.63 | 4hwb_HL | 0.39 | 4y5x_GH | 1.18 | 5o1r_HL | 0.50 |
| 1n8z_BA | 0.45 | 3i50_HL | 0.94 | 4i18_AB | 0.51 | 4y5y_AB | 0.70 | 5o4g_BA | 0.77 |
| 1nby_BA | 0.17 | 3idx_HL | 0.02 | 4i18_HL | 0.51 | 4ybl_BC | 1.10 | 5o6v_HL | 1.50 |
| 1nbz_BA | 0.20 | 3idy_BC | 0.37 | 4i2x_BA | 0.01 | 4ybq_DC | 0.39 | 5ob5_HL | 0.03 |
| 1nca_HL | 0.05 | 3iu3_HL | 1.22 | 4i2x_DC | 0.24 | 4yc2_BC | 1.05 | 5occ_HL | 0.02 |
| 1ncb_HL | 0.05 | 3iyw_HL | 0.02 | 4i3r_HL | 0.45 | 4yc2_HL | 0.93 | 5sx4_HL | 0.22 |
| 1ncc_HL | 0.40 | 3j5m_HG | 0.73 | 4i3s_HL | 0.54 | 4ydi_HL | 0.76 | 5sx5_HL | 0.16 |
| 1ndg_BA | 0.01 | 3j70_MN | 0.80 | 4i77_HL | 0.03 | 4ydj_HL | 0.05 | 5sy8_HL | 0.03 |
| 1ndm_BA | 0.01 | 3jwo_HL | 0.39 | 4idj_HL | 0.70 | 4ydk_HL | 0.03 | 5t33_HL | 0.72 |
| 1nfd_FE | 0.55 | 3k2u_HL | 0.03 | 4ij3_CB | 0.48 | 4ydl_BC | 0.08 | 5t3s_HL | 0.41 |
| 1nfd_HG | 0.44 | 3kj4_HL | 0.74 | 4iof_EF | 0.61 | 4ye4_HL | 0.79 | 5t3x_DE | 1.09 |
| 1nma_HL | 0.57 | 3kj6_HL | 1.17 | 4irz_HL | 0.47 | 4yfl_FI | 1.87 | 5t3z_DE | 1.01 |
| 1nmb_HL | 0.01 | 3klh_DC | 0.79 | 4j4p_HL | 1.23 | 4yfl_HL | 1.69 | 5t3z_HL | 0.83 |
| 1nsn_HL | 1.16 | 3kr3_HL | 0.03 | 4j6r_HL | 0.03 | 4yk4_CB | 0.45 | 5t5b_AB | 0.45 |
| 1oak_HL | 0.03 | 3ks0_KJ | 0.56 | 4jan_HL | 1.01 | 4ypg_HL | 0.54 | 5t5f_HL | 0.76 |
| 1oaz_HL | 0.45 | 3l5w_HL | 0.51 | 4jb9_HL | 0.34 | 4ywg_HL | 0.38 | 5t6l_HL | 0.27 |
| 1ob1_BA | 0.67 | 3l5x_HL | 0.04 | 4jdt_HL | 1.28 | 4yx2_HL | 0.22 | 5t80_HL | 0.33 |
| 1orq_BA | 0.50 | 3l95_HL | 1.39 | 4jhw_HL | 0.27 | 4yxh_HL | 0.19 | 5t85_HL | 0.29 |
| 1ors_BA | 0.01 | 3ldb_CB | 0.31 | 4jkp_HL | 0.28 | 4yxk_HL | 0.29 | 5te4_HL | 0.32 |
| 1osp_HL | 0.06 | 3lh2_KO | 0.35 | 4jlr_HL | 0.27 | 4yxl_HL | 0.27 | 5te6_HL | 0.26 |
| 1p2c_BA | 0.17 | 3lhp_HL | 0.39 | 4jm2_DC | 0.30 | 4yzf_EF | 0.71 | 5te7_HL | 0.02 |
| 1pkq_GF | 0.66 | 3liz_HL | 0.02 | 4jpk_HL | 0.64 | 4z5r_ZY | 0.48 | 5tfw_HL | 0.03 |
| 1qfu_HL | 0.55 | 3lqa_HL | 0.63 | 4jpv_HL | 0.99 | 4zfg_HL | 0.02 | 5th9_IM | 0.68 |
| 1qfw_IM | 0.58 | 3lzf_HL | 0.55 | 4jpw_HL | 1.14 | 4zpt_AB | 0.28 | 5thr_NK | 1.07 |
| 1qle_HL | 0.62 | 3ma9_HL | 0.02 | 4jr9_HL | 0.41 | 4zpt_HL | 0.40 | 5thr_TU | 1.78 |
| 1rjl_BA | 0.53 | 3mac_HL | 0.38 | 4jre_HL | 0.42 | 4zpv_HL | 0.46 | 5tih_HL | 0.02 |
| 1rzk_HL | 0.44 | 3mj9_HL | 0.80 | 4jzj_HL | 0.89 | 4zs6_HL | 0.67 | 5tl5_HL | 0.02 |
| 1s78_FE | 0.49 | 3mxw_HL | 0.03 | 4k24_HL | 0.86 | 4zs7_HL | 0.76 | 5tlj_BA | 0.68 |
| 1sy6_HL | 0.02 | 3n85_HL | 0.78 | 4k2u_HL | 0.23 | 4zso_DC | 0.18 | 5tlj_DC | 0.51 |
| 1tzh_BA | 0.44 | 3ncy_PS | 0.86 | 4k3j_HL | 0.96 | 4zxb_AB | 0.96 | 5tlk_BA | 0.25 |
| 1ua6_HL | 0.20 | 3nfp_HL | 0.96 | 4k8r_DC | 0.58 | 4zxb_CD | 0.57 | 5tlk_DC | 0.39 |
| 1uac_HL | 0.45 | 3ngb_BC | 0.40 | 4k8r_HL | 0.27 | 4zyp_KM | 0.47 | 5tpn_HL | 0.87 |
| 1uj3_BA | 0.01 | 3nh7_HL | 0.72 | 4k9e_HL | 0.91 | 4zyp_NO | 0.44 | 5tpw_HL | 0.93 |
| 1v7m_HL | 0.46 | 3nid_HL | 0.24 | 4kht_HL | 0.43 | 5a7x_HG | 0.40 | 5tq0_HL | 0.90 |
| 1v7n_IM | 0.62 | 3nps_BC | 0.03 | 4khx_HL | 0.39 | 5aco_HL | 0.65 | 5tq2_HL | 0.96 |
| 1vfb_BA | 0.26 | 3o2d_HL | 0.03 | 4ki5_EF | 0.64 | 5anm_BA | 0.26 | 5tqq_HL | 0.78 |
| 1wej_HL | 0.29 | 3ogc_AB | 0.37 | 4kro_DC | 0.39 | 5b3j_HL | 1.54 | 5tr1_IM | 0.81 |
| 1xcq_DC | 0.00 | 3opz_HL | 0.78 | 4krp_DC | 0.36 | 5b71_DC | 0.29 | 5tru_hl | 0.60 |
| 1xct_BA | 0.00 | 3p0y_HL | 0.02 | 4kv5_JI | 0.41 | 5b8c_BA | 0.24 | 5tud_CB | 0.69 |
| 1xct_DC | 0.00 | 3p11_HL | 0.47 | 4kvn_HL | 0.63 | 5b8c_HG | 0.15 | 5tz2_HL | 0.02 |
| 1xf5_BA | 0.00 | 3pgf_HL | 0.47 | 4kxz_JI | 0.47 | 5bo1_HL | 0.61 | 5tzu_HL | 0.02 |
| 1xgq_BA | 0.01 | 3pjs_BA | 0.77 | 4l5f_HL | 0.02 | 5bv7_CB | 0.12 | 5u7o_HL | 0.67 |
| 1xgr_BA | 0.01 | 3pnw_BA | 0.51 | 4leo_AB | 0.81 | 5bv7_HL | 0.02 | 5u8q_HL | 0.83 |
| 1xgt_BA | 0.15 | 3q1s_HL | 0.03 | 4lf3_ED | 0.43 | 5bvp_HL | 0.46 | 5u8r_HL | 0.83 |
| 1xiw_HG | 0.21 | 3q3g_DC | 0.61 | 4liq_HL | 0.74 | 5c0r_HL | 0.97 | 5ucb_HL | 0.02 |
| 1yjd_HL | 0.53 | 3qa3_BA | 0.61 | 4lmq_HL | 0.63 | 5c0s_HL | 0.23 | 5udc_HL | 0.35 |
| 1ymh_DC | 0.00 | 3qum_BA | 1.16 | 4lqf_HL | 0.03 | 5c6t_HL | 1.05 | 5uea_HL | 0.81 |
| 1ynt_BA | 0.48 | 3qum_KM | 0.91 | 4lsp_HL | 0.22 | 5c7x_HL | 0.43 | 5uek_HL | 0.02 |
| 1ynt_DC | 0.48 | 3qwo_HL | 0.02 | 4lsq_HL | 0.26 | 5c8j_AB | 0.79 | 5uem_HL | 0.68 |
| 1yqv_HL | 0.03 | 3r08_HL | 0.62 | 4lsr_HL | 0.41 | 5cba_AB | 0.01 | 5ug0_DC | 0.91 |
| 1yy9_DC | 0.40 | 3r1g_HL | 0.52 | 4lss_HL | 0.38 | 5cbe_CD | 1.21 | 5ugy_HL | 0.55 |
| 1yyl_RQ | 0.37 | 3raj_HL | 0.83 | 4lst_HL | 0.33 | 5cd5_CD | 0.64 | 5ukr_HL | 0.50 |
| 1yym_RQ | 0.27 | 3rhw_FN | 0.66 | 4lsu_HL | 0.03 | 5cez_HL | 0.52 | 5um8_HL | 0.71 |
| 1z3g_HL | 0.83 | 3rkd_HL | 0.23 | 4lsv_HL | 1.00 | 5cjo_HL | 0.72 | 5umn_EF | 0.22 |
| 1za3_HL | 1.02 | 3ru8_HL | 0.36 | 4lu5_HL | 1.44 | 5cjq_HL | 0.60 | 5uoe_PQ | 0.58 |
| 1ztx_HL | 0.03 | 3rvw_DC | 1.17 | 4lvh_BC | 1.31 | 5cus_JN | 1.01 | 5ush_HL | 0.33 |
| 2a0l_FE | 0.65 | 3s35_HL | 0.03 | 4lvn_CB | 0.27 | 5czv_HL | 0.59 | 5usl_HL | 1.46 |
| 2adf_HL | 0.02 | 3s36_HL | 0.50 | 4lvo_CB | 0.01 | 5czx_HL | 0.36 | 5utz_FG | 1.24 |
| 2aep_HL | 0.02 | 3s37_HL | 0.46 | 4m1c_CD | 0.71 | 5d1q_BA | 0.51 | 5v2a_HL | 1.26 |
| 2aeq_HL | 0.61 | 3sdy_HL | 0.61 | 4m1g_HL | 0.02 | 5d1q_CD | 0.64 | 5v7j_HL | 1.21 |
| 2arj_HL | 0.97 | 3se8_HL | 0.02 | 4m5z_HL | 0.02 | 5d1x_BA | 0.52 | 5v8l_HL | 0.00 |
| 2atk_AB | 0.29 | 3se9_HL | 0.04 | 4m62_IM | 0.01 | 5d1x_CD | 1.14 | 5v8m_SU | 0.00 |
| 2b2x_HL | 0.17 | 3skj_HL | 0.95 | 4m7l_HL | 0.55 | 5d1z_DC | 0.25 | 5vag_CB | 0.01 |
| 2b4c_HL | 0.92 | 3sob_HL | 0.64 | 4m8q_AB | 0.52 | 5d1z_GH | 0.91 | 5veb_AB | 0.02 |
| 2bdn_HL | 0.81 | 3sqo_HL | 0.70 | 4m8q_HL | 0.45 | 5d72_MN | 0.42 | 5vgj_HL | 0.43 |
| 2cmr_HL | 0.03 | 3t2n_HL | 0.65 | 4ma7_HL | 0.27 | 5d8j_HL | 0.91 | 5vic_HL | 0.72 |
| 2dd8_HL | 0.02 | 3t3m_EF | 0.18 | 4mhh_HL | 0.63 | 5d93_CB | 0.01 | 5vig_HL | 0.76 |
| 2dqc_HL | 0.13 | 3tt1_IM | 0.62 | 4mhj_HL | 0.79 | 5d96_CB | 0.01 | 5vjo_AB | 0.35 |
| 2dqe_HL | 0.13 | 3tt3_HL | 0.64 | 4mwf_AB | 0.92 | 5d9q_FE | 0.53 | 5vjq_CD | 0.43 |
| 2dqf_BA | 0.48 | 3u2s_HL | 0.69 | 4mxv_HL | 0.65 | 5dfv_CD | 0.56 | 5vkd_HL | 0.61 |
| 2dqg_HL | 0.18 | 3u30_CB | 0.42 | 4mxw_HL | 0.73 | 5dhv_AB | 0.13 | 5vke_AB | 0.20 |
| 2dqj_HL | 0.13 | 3u4e_HL | 0.61 | 4nhh_MK | 0.25 | 5dhz_HL | 0.04 | 5vl3_HL | 0.53 |
| 2eiz_BA | 0.01 | 3u7y_HL | 0.04 | 4nnp_HL | 0.86 | 5dmi_HL | 0.43 | 5vl7_HL | 1.09 |
| 2fd6_HL | 0.03 | 3u9p_HL | 1.01 | 4np4_HL | 0.58 | 5do2_HL | 0.02 | 5vlp_HL | 1.02 |
| 2fjh_HL | 0.71 | 3u9u_AB | 0.62 | 4np4_IM | 0.56 | 5dum_HL | 0.79 | 5vn3_MO | 0.93 |
| 2h9g_HL | 1.43 | 3uaj_HL | 1.12 | 4nzt_HL | 0.56 | 5dup_HL | 0.84 | 5vob_HL | 0.99 |
| 2i5y_RQ | 0.39 | 3ubx_HL | 0.88 | 4o02_HL | 0.72 | 5dur_HL | 1.33 | 5voc_HL | 1.01 |
| 2i9l_FE | 0.46 | 3uc0_HL | 0.94 | 4o58_HL | 0.37 | 5dwu_HL | 1.31 | 5vpl_DC | 1.08 |
| 2iff_HL | 0.36 | 3ulu_DC | 1.16 | 4o5i_WX | 0.25 | 5e1a_AB | 0.35 | 5vta_HL | 0.74 |
| 2j6e_HL | 0.86 | 3ulu_FE | 0.54 | 4o9h_HL | 0.03 | 5e8e_BA | 1.13 | 5vyf_HL | 0.78 |
| 2j88_HL | 0.98 | 3ulu_HL | 0.43 | 4od2_BA | 0.50 | 5e94_BA | 1.14 | 5w06_HL | 0.44 |
| 2jel_HL | 0.03 | 3ulv_DC | 1.19 | 4odx_HB | 0.31 | 5eii_HL | 0.61 | 5w08_KL | 0.73 |
| 2jix_DG | 1.54 | 3ulv_FE | 0.48 | 4oga_CD | 1.22 | 5en2_AB | 0.03 | 5w0d_BC | 0.51 |
| 2jix_FA | 0.99 | 3ulv_HL | 0.53 | 4ogx_HL | 0.03 | 5eu7_FD | 0.51 | 5w0k_EF | 0.52 |
| 2ltq_CB | 0.57 | 3v4p_HL | 1.40 | 4ogy_HL | 0.27 | 5ezo_HL | 0.35 | 5w0k_HL | 0.59 |
| 2nr6_FE | 0.51 | 3v4v_HL | 1.65 | 4ogy_MN | 0.27 | 5f3b_EF | 0.09 | 5w1k_LK | 0.76 |
| 2ny3_DC | 0.12 | 3v6o_DF | 0.42 | 4oii_HL | 0.58 | 5f3h_CD | 0.78 | 5w1k_ON | 0.58 |
| 2ny4_DC | 0.13 | 3v6z_AB | 0.74 | 4okv_AB | 0.02 | 5f6j_HF | 0.54 | 5w1m_DC | 0.34 |
| 2ny5_HL | 0.22 | 3v7a_EH | 1.05 | 4olu_HL | 0.21 | 5f96_HL | 0.04 | 5w23_HL | 0.73 |
| 2ny7_HL | 0.29 | 3vg9_CB | 0.80 | 4olv_HL | 0.20 | 5f9o_HL | 0.03 | 5w2b_HL | 0.59 |
| 2nz9_DC | 0.52 | 3vga_CB | 0.84 | 4olw_HL | 0.19 | 5f9w_HL | 0.63 | 5w3e_EG | 0.67 |
| 2oz4_HL | 0.78 | 3vi3_HL | 0.77 | 4olx_HL | 0.03 | 5fb8_BA | 0.01 | 5w3l_EG | 0.66 |
| 2q8a_HL | 0.02 | 3vi4_HL | 0.76 | 4oly_HL | 0.18 | 5fcu_HL | 0.47 | 5w3m_EG | 0.65 |
| 2q8b_HL | 0.03 | 3vrl_HL | 0.52 | 4olz_HL | 0.15 | 5fec_HL | 0.41 | 5w3o_DE | 0.75 |
| 2qad_DC | 0.88 | 3w11_CD | 1.23 | 4om0_HL | 0.04 | 5fhc_HL | 0.17 | 5w42_HL | 0.89 |
| 2qqk_HL | 0.95 | 3w2d_HL | 0.71 | 4om1_HL | 0.16 | 5fyl_DE | 0.77 | 5w5x_HL | 0.27 |
| 2qql_HL | 0.93 | 3w9e_AB | 0.73 | 4oqt_HL | 1.04 | 5fyl_HL | 0.74 | 5w5z_HL | 0.03 |
| 2qqn_HL | 0.02 | 3wd5_HL | 0.67 | 4ot1_HL | 0.03 | 5g64_HL | 0.84 | 5w6d_HL | 0.57 |
| 2qr0_RQ | 0.52 | 3wfb_HL | 0.22 | 4p59_HL | 0.61 | 5ggr_HL | 0.67 | 5w6g_HL | 0.45 |
| 2r0k_HL | 0.85 | 3wih_HL | 0.51 | 4plj_HL | 0.03 | 5ggs_CD | 0.29 | 5w9h_EF | 0.91 |
| 2r0l_HL | 0.54 | 3wkm_HL | 0.03 | 4plk_HL | 0.47 | 5ggt_HL | 1.02 | 5w9i_KL | 0.79 |
| 2r29_HL | 1.20 | 3wlw_HL | 0.90 | 4pp1_DC | 1.49 | 5ggv_HL | 0.02 | 5w9j_BC | 1.17 |
| 2r4r_HL | 1.19 | 3wxv_HL | 0.39 | 4pp2_DC | 0.35 | 5gjs_HL | 1.06 | 5w9k_HI | 0.90 |
| 2r4s_HL | 1.16 | 3wxw_HL | 0.19 | 4ps4_HL | 0.31 | 5gjt_HL | 1.01 | 5w9l_HI | 0.81 |
| 2r56_HL | 0.92 | 3x3f_HL | 0.03 | 4py8_IJ | 0.66 | 5gmq_BC | 0.96 | 5w9m_BC | 0.93 |
| 2r69_HL | 1.39 | 3zdy_EF | 0.22 | 4q6i_HL | 1.14 | 5grj_HL | 1.16 | 5w9n_BC | 0.91 |
| 2uzi_HL | 0.02 | 3zdz_EF | 0.20 | 4qci_HL | 0.17 | 5gs0_DC | 0.56 | 5w9o_BC | 0.95 |
| 2vdp_HL | 0.25 | 3ze0_HL | 0.22 | 4qex_HL | 0.83 | 5gzo_HL | 0.76 | 5w9p_DE | 0.83 |
| 2vir_BA | 0.54 | 3ze1_HL | 0.25 | 4qhu_HL | 0.42 | 5h35_HI | 0.41 | 5wb9_HL | 0.24 |
| 2vis_BA | 0.54 | 3ze2_HL | 0.24 | 4qti_HL | 0.46 | 5h37_GH | 1.22 | 5wt9_HL | 0.65 |
| 2vit_BA | 0.54 | 3zkm_HL | 0.12 | 4qww_DC | 0.32 | 5hbt_DC | 0.52 | 5wux_HL | 0.47 |
| 2vwe_EC | 1.34 | 3zkn_HL | 0.14 | 4r0l_HL | 0.72 | 5hbv_DC | 0.19 | 5x0t_AB | 0.12 |
| 2vxq_HL | 0.02 | 3ztj_GH | 1.03 | 4r8w_HL | 0.32 | 5hdq_HL | 0.03 | 5x8l_GL | 1.54 |
| 2vxt_HL | 0.39 | 4aei_HL | 0.25 | 4rau_BA | 0.41 | 5hhv_HL | 0.37 | 5x8m_BC | 0.37 |
| 2w9e_HL | 0.39 | 4ag4_HL | 1.05 | 4rdq_GF | 1.00 | 5hj3_MN | 0.39 | 5xbm_BA | 0.81 |
| 2wub_RQ | 0.39 | 4al8_HL | 0.03 | 4rfn_HL | 0.64 | 5hys_AB | 0.02 | 5xez_HL | 0.67 |
| 2wuc_HL | 0.47 | 4ala_HL | 0.34 | 4rfo_HL | 0.58 | 5i5k_HL | 1.71 | 5xf1_HL | 0.65 |
| 2x7l_GI | 0.40 | 4bz1_HL | 0.02 | 4rgm_CB | 0.97 | 5i8h_IJ | 0.69 | 5xhv_HL | 1.28 |
| 2xqb_HL | 1.00 | 4bz2_HL | 0.03 | 4rgm_HL | 0.51 | 5i9q_HL | 0.79 | 5xj3_JK | 0.63 |
| 2xqy_GL | 0.03 | 4cad_ED | 0.39 | 4rgn_BC | 0.33 | 5ies_HL | 0.63 | 5xj4_HL | 0.01 |
| 2xra_HL | 0.03 | 4ckd_HL | 0.11 | 4rgn_DE | 1.24 | 5if0_AB | 0.60 | 5xjm_HL | 0.48 |
| 2xtj_DB | 0.43 | 4cmh_BC | 0.03 | 4rgo_HL | 0.03 | 5igx_HL | 0.54 | 5xku_CB | 0.02 |
| 2xwt_AB | 0.03 | 4cni_HL | 0.15 | 4rqs_DC | 0.36 | 5ikc_BA | 0.01 | 5xmh_HL | 0.66 |
| 2yc1_AB | 0.05 | 4d3c_HL | 0.66 | 4rrp_HB | 0.56 | 5j13_CB | 0.06 | 5xs7_HL | 0.94 |
| 2ypv_HL | 0.02 | 4d9q_HL | 0.27 | 4rwy_HL | 0.04 | 5j3h_CD | 1.06 | 5xwd_HD | 0.67 |
| Testing dataset | | | | | | | | | |
| 5bjz_DH | 0.58 | 6b0s_HL | 0.31 | 6ck9_HL | 0.57 | 6fax_HL | 0.64 | 6mek_BD | 0.59 |
| 5bk1_HL | 0.52 | 6b3s_HL | 0.55 | 6cm3_ON | 0.60 | 6feq_FE | 0.58 | 6mek_FE | 0.41 |
| 5bk2_HL | 1.00 | 6b70_EF | 0.95 | 6cm3_RS | 1.17 | 6fgb_HL | 0.94 | 6mft_HL | 0.03 |
| 5otj_HL | 0.02 | 6b7z_EF | 1.41 | 6cmg_CB | 0.66 | 6fla_HL | 0.27 | 6mhr_AB | 0.59 |
| 5wdu_DE | 0.43 | 6b9j_HL | 0.67 | 6cmi_DC | 0.67 | 6flb_HL | 0.02 | 6mi2_AB | 0.44 |
| 5whk_HL | 0.02 | 6bae_BA | 0.13 | 6cmo_HL | 1.50 | 6flc_HL | 0.16 | 6ml8_HL | 0.39 |
| 5wi9_HL | 0.62 | 6bah_BA | 0.09 | 6cnv_HL | 0.49 | 6fn1_CB | 0.74 | 6mlk_HL | 0.04 |
| 5wob_UV | 0.67 | 6bck_HL | 1.43 | 6crq_DE | 0.88 | 6fn4_CB | 0.91 | 6mlm_EI | 1.01 |
| 5xxy_HL | 1.24 | 6bdz_HL | 0.74 | 6cse_HL | 0.80 | 6fxn_DE | 0.40 | 6mto_HL | 0.58 |
| 5y11_AB | 0.02 | 6bf7_EF | 1.24 | 6csf_HL | 0.80 | 6fy1_HL | 0.45 | 6mtq_HL | 0.57 |
| 5y2l_IJ | 0.57 | 6bf9_CD | 1.25 | 6cue_QR | 0.91 | 6gv1_HL | 0.83 | 6mu6_HL | 0.21 |
| 5y9j_HL | 0.02 | 6bfq_HL | 0.70 | 6cuf_mn | 0.89 | 6h3t_IM | 0.75 | 6mu7_DE | 0.27 |
| 5yoy_PM | 0.39 | 6bfs_HL | 0.02 | 6cuf_qr | 0.68 | 6h3u_HL | 0.69 | 6mu7_HL | 0.19 |
| 5ywy_HL | 0.54 | 6bft_AB | 0.33 | 6cw2_AB | 0.81 | 6h5n_CB | 0.85 | 6mu8_HL | 0.23 |
| 5yy5_HL | 1.10 | 6bft_HL | 0.29 | 6cw3_AB | 0.60 | 6hco_DC | 0.61 | 6muf_HL | 0.38 |
| 5zxv_HL | 1.02 | 6bgt_BA | 0.44 | 6cwt_CD | 0.70 | 6hf1_CB | 0.17 | 6mug_HL | 0.44 |
| 6a0z_HL | 0.84 | 6bit_JL | 0.03 | 6cxy_HL | 1.06 | 6i04_HL | 0.93 | 6mui_CD | 0.54 |
| 6a3w_AB | 0.02 | 6bkb_HL | 0.71 | 6cyf_DC | 0.43 | 6i8s_HL | 1.09 | 6mw9_CD | 0.54 |
| 6a4k_IM | 1.07 | 6bkc_HL | 1.12 | 6d0u_HI | 0.35 | 6i9i_HL | 0.02 | 6mwc_CD | 0.54 |
| 6a67_HL | 0.18 | 6bkd_HL | 0.98 | 6d2p_HL | 0.94 | 6iea_HL | 0.04 | 6mwv_KL | 0.54 |
| 6a78_HL | 0.02 | 6bp2_HL | 0.62 | 6d6t_JI | 0.81 | 6ieb_HL | 0.03 | 6mwx_KL | 0.54 |
| 6al5_HL | 0.37 | 6bpa_BC | 0.68 | 6d6u_JI | 0.82 | 6iec_HL | 0.67 | 6mwx_OP | 0.54 |
| 6aod_BA | 0.31 | 6bpc_BC | 0.68 | 6ddm_BA | 0.01 | 6iek_BC | 1.03 | 6myy_GF | 0.41 |
| 6apb_HL | 0.47 | 6bpe_EF | 1.03 | 6ddr_BA | 0.01 | 6ii4_HL | 0.92 | 6nb3_HL | 0.97 |
| 6apd_FG | 0.56 | 6c5v_HL | 0.68 | 6ddv_BA | 0.06 | 6ii8_HL | 1.03 | 6nm6_HL | 0.37 |
| 6apd_JL | 0.49 | 6c6y_HL | 0.67 | 6de7_HL | 0.79 | 6ii9_MN | 1.13 | 6nm6_UV | 0.45 |
| 6aq7_HL | 0.03 | 6c6z_HL | 0.02 | 6dfi_HL | 0.03 | 6iut_HL | 0.02 | 6nn3_HL | 1.04 |
| 6aru_CB | 0.37 | 6c9u_HL | 0.03 | 6dfj_HL | 0.28 | 6iuv_CD | 0.03 | 6nnf_HL | 0.43 |
| 6att_HL | 0.62 | 6cbv_HL | 0.67 | 6did_KE | 0.07 | 6ivz_HL | 0.01 | 6nnf_UV | 0.47 |
| 6ayz_BD | 0.55 | 6cde_MN | 1.00 | 6e3h_HL | 0.98 | 6iw2_KL | 0.28 | 6nnj_UV | 0.36 |
| 6az2_AF | 0.52 | 6cde_QR | 0.65 | 6e62_BC | 0.67 | 6j5d_HL | 0.15 | 6o39_BA | 0.45 |
| 6azz_CB | 0.05 | 6cdi_QR | 0.56 | 6e63_HL | 0.29 | 6j5g_HL | 0.25 | 6o3a_BA | 0.43 |
| 6b08_CB | 0.08 | 6ce0_DE | 0.47 | 6eay_HL | 0.95 | 6mar_MN | 1.35 | 6o3b_GE | 0.51 |
| 6b0a_HL | 0.02 | 6cf2_AB | 0.43 | 6edu_LM | 1.13 | 6mdt_DE | 0.51 | 6qee_CB | 0.86 |
| 6b0e_BA | 0.51 | 6ch7_DE | 1.10 | 6edu_PQ | 1.27 | 6mdt_HL | 0.82 | 6qex_CB | 0.69 |
| 6b0g_DC | 0.08 | 6chb_DE | 1.72 | 6elu_EF | 0.03 | 6meh_HL | 0.02 | 6r2s_BA | 0.45 |
| 6b0h_DC | 0.43 | 6chb_JK | 1.45 | 6eti_FE | 0.42 | 6mei_HL | 0.42 | 6r8x_CB | 0.15 |
| 6b0n_DE | 0.80 | 6chb_MN | 1.60 | 6ewb_GI | 0.72 | 6mej_AB | 0.40 |  |  |
| 6b0n_HL | 0.64 | 6chb_OP | 2.04 | 6eyo_HL | 0.56 | 6mej_HL | 0.55 |  |  |

^a^ Each score representing the RMSD value between crystalized antibody structure and modelled structure with the same PDB id.

**Supplementary Methods**

**Supplementary Method 1. Surface patch generation and score prediction**

For an input antigen, SEPPA-mAb will automatically generalize the spatial patch by setting each surface residue as patch center on antigen protein, and paired with the CDR patch of the corresponding antibody. Surface residues were those with more than 1Å^2^ solvent accessible surface areas (SASA) calculated by Naccess V2.1.1 (1). For each surface residue, the surface patch was extracted by its neighboring surface residues within 10Å.

To get the patch complementarity (PC) scores, SEPPA-mAb will first generate the structure fingerprints for both antigen patches and the CDRs patch by considering physic-chemical and structural features. Then, the PC score for each antigen patch can be calculated through an appropriate machine-learning algorithm.

A series of fingerprints were designed to describe surface patches and CDRs using both structure layout and physic-chemical properties. Structure and physicochemical properties such as structural composition, electrostatic interactions, hydrophobic interactions, hydrogen-bond and Van der Waals forces which play essential roles in the specific binding of antigen-antibody interactions were taken into consideration (2-5). Thus, 8 properties including ZIMJ680104_acid, ZIMJ680104_base, FAUJ880109, ARGP820101, FAUJ880103, GEIM800101, CHOP780101, and DAYM780101 were selected from AAindex database (5). Then, the cylinder structure model was established to describe the surface patch and CDRs. For each surface patch on antigen, a minimum external cylinder with a 10 Å rotating radius and 20 Å center axis was generated. Specifically, by setting a vector through the geometric center of antigen and patch center as center axis, along with a 10 Å radius rotating plane revolving around the center axis, each of the surface residues can be punched into the certain position of the cylinder model. After setting the radius pixel as 2 Å and center axis pixel as 4 Å, a 2-dimensional grid that contains 25 (10/2 * 20/4) bits was screened. Finally, a 200 (25*8) bit fingerprints were generated to describe the surface patch.

The CDR patch and fingerprint were generated by the same cylinder model with slight modifications on defining two calibration points to generate the cylinder axis. One calibration point is defined as the geometric center of four residues from two-disulfide bonds (light chain: A23–A88; heavy chain: B22–B92) in the Fv region, and the other point is defined as the geometric center of all residues in CDR patch.

Briefly, for each complex in the training dataset, one patch was derived from antibody side (CDR patch), and 20 surface patches were chosen from antigen side. After pairing between the CDR patch and 20 antigen patches, 20 pairs of patch from each complex were used for model training.

In detail of Ab side, the CDR patch was described via the cylinder model based on two centers described as above. From antigen side, for each surface residue ${Res}_{r}$ of antigen, a surface patch was generated covering its neighboring residues within 10 Å, as formula (S1) illustrated:

${Res}_{i}\in{Patch}_{r}, if D_{min}({Res}_{i}, {Res}_{r})\leq10Å$ (S1)

Where$D_{min}({Res}_{i}, {Res}_{r})$ illustrated minimum atom distance between any residue ${Res}_{i}$ and the target surface residue ${Res}_{r}$.

For an antigen with *N* surface residues, *N* patches will be generated. Then, all *N* surface patches were ranked according to the overlapping extent with true epitope residues. Top 10 patches containing the most epitope residues were defined as the positive patches, and 10 random patches with no epitope residues were defined as the negative ones. After pairing with the CDR patch, 10 positive and 10 negative samples can be generated for each antigen-antibody complex. Worth note that, all positive patches in training have at least 50% overlapping with true epitope residues.

By combining both patch fingerprints and CDRs fingerprints, 400-bit fingerprints were generated to predict the potential interaction probability between the surface patch and CDRs. To divide surface patch as positive sample or negative sample, we first calculated target value (proportion of residues in the patch that are part of real epitope) for each patch. Then, for each antigen in the training dataset, the top 10 patches with a target value above 0.5 were defined as positive sample, and 10 patches were random chosen as negative sample whose target value was 0. Furthermore, 8 different machine learning approaches, including XGBoost (XGB), Support Vector Machine (SVM), Random Forrest (RF), Decision Tree (DT), Multi-Layer Perceptron (MLP), Gradient Descent (SGD), Gaussian Naïve Bayes (GNB), and Linear Regression (LR) were introduced to predict the PC score for surface patch based on the previously constructed fingerprints.

**Supplementary Method 2. Epitope prediction by molecular docking algorithms**

Two docking methods were introduced in this work: ZDOCK (6) and ClusPro (7). For each antigen and its corresponding antibody in the 193 testing dataset, antibody-antigen docking was performed by providing information about the binding interface which includes all surface residues of the antigen and CDRs of the antibody. ZDOCK (6) predictions were obtained using version 3.0.2. The sampling was set to 2000 models. ZDOCK (6) allows the user to assign highly unfavorable contact energy to the residues which are known not to be involved in the binding. Accordingly, non-surface residues of the antigen and non-CDRs of the antibody were blocked. The ClusPro webserver (https://cluspro.org) was used in the Antibody Mode using default settings. ClusPro (7) offers automated masking of non-CDR regions for antibodies. For each antigen and its corresponding antibody, only the top 10 ranked solutions by the two docking methods were analyzed.

To evaluate the performance of docking software on epitope prediction, the AUC value was calculated for each antigen. The residues in the interface region of generated models were assigned a score of 1 and others were assigned 0. Then, for each antigen, the AUC value for epitope prediction from generated antibody-antigen models can be calculated. The AUC value of top *N* ranked solutions is the average AUC value of the top *N* ranked solutions.

**Supplementary Method 3. Antibody homologous modeling**

Since the antibody structures might not be always available for prediction, we tested the model robustness on homology modeled Ab structures. Tool of ABodyBuilder (8), a fully automated Ab structure prediction server, was chosen for all the Abs in our test dataset by input their sequences. The RMSD between crystalized antibody structures and modelled antibody structures were listed in **Table S7**.

**References**

1. Hubbard, S. and Thornton, J. (1996) NACCESS v. 2.1. 1-Atomic Solvent Accessible Area Calculations. *Department of biochemistry and molecular biology*.

2. Lo Conte, L., Chothia, C. and Janin, J. (1999) The atomic structure of protein-protein recognition sites. *J Mol Biol*, **285**, 2177-2198.

3. Shulman-Peleg, A., Shatsky, M., Nussinov, R. and Wolfson, H.J. (2007) Spatial chemical conservation of hot spot interactions in protein-protein complexes. *Bmc Biology*, **5**.

4. Sela-Culang, I., Alon, S. and Ofran, Y. (2012) A Systematic Comparison of Free and Bound Antibodies Reveals Binding-Related Conformational Changes. *Journal of Immunology*, **189**, 4890-4899.

5. Kawashima, S., Pokarowski, P., Pokarowska, M., Kolinski, A., Katayama, T. and Kanehisa, M. (2008) AAindex: amino acid index database, progress report 2008. *Nucleic Acids Res*, **36**, D202-205.

6. Pierce, B.G., Wiehe, K., Hwang, H., Kim, B.H., Vreven, T. and Weng, Z. (2014) ZDOCK server: interactive docking prediction of protein-protein complexes and symmetric multimers. *Bioinformatics*, **30**, 1771-1773.

7. Kozakov, D., Hall, D.R., Xia, B., Porter, K.A., Padhorny, D., Yueh, C., Beglov, D. and Vajda, S. (2017) The ClusPro web server for protein-protein docking. *Nat Protoc*, **12**, 255-278.

8. Leem, J., Dunbar, J., Georges, G., Shi, J. and Deane, C.M. (2016), *MAbs*. Taylor & Francis, Vol. 8, pp. 1259-1268.
